# Severe bacterial neonatal infections in Madagascar, Senegal, and Cambodia: A multicentric community-based cohort study

**DOI:** 10.1371/journal.pmed.1003681

**Published:** 2021-09-28

**Authors:** Bich-Tram Huynh, Elsa Kermorvant-Duchemin, Rattanak Chheang, Frederique Randrianirina, Abdoulaye Seck, Elisoa Hariniaina Ratsima, Zafitsara Zo Andrianirina, Jean-Baptiste Diouf, Armya Youssouf Abdou, Sophie Goyet, Véronique Ngo, Siyin Lach, Long Pring, Touch Sok, Michael Padget, Fatoumata Diene Sarr, Laurence Borand, Benoit Garin, Jean-Marc Collard, Perlinot Herindrainy, Agathe de Lauzanne, Muriel Vray, Elisabeth Delarocque-Astagneau, Didier Guillemot, On behalf of the BIRDY study group

**Affiliations:** 1 Université Paris-Saclay, UVSQ, Inserm, CESP, Anti-infective Evasion and Pharmacoepidemiology Team, Montigny-Le-Bretonneux, France; 2 Institut Pasteur, Epidemiology and Modelling of Antibiotic Evasion (EMAE), Paris, France; 3 AP-HP, Hôpital Necker-Enfants Malades, Department of Neonatal Medicine, Université de Paris, Paris, France; 4 Medical Biology Unit, Institut Pasteur du Cambodge, Phnom Penh, Cambodia; 5 Centre de Biologie Clinique, Institut Pasteur de Madagascar, Antananarivo, Madagascar; 6 Laboratory of Medical Biology, Institut Pasteur de Dakar, Dakar, Senegal; 7 Pediatric Ward, Centre Hospitalier de Soavinandriana, Antananarivo, Madagascar; 8 Centre Hospitalier Roi Baudouin, Guédiawaye, Dakar, Sénégal; 9 Epidemiology and Public Health Unit, Institut Pasteur du Cambodge, Phnom Penh, Cambodia; 10 Ministry of Health, Phnom Penh, Cambodia; 11 Epidemiology of Infectious Diseases Unit, Institut Pasteur de Dakar, Dakar, Senegal; 12 Experimental Bacteriology Unit, Institut Pasteur de Madagascar, Antananarivo, Madagascar; 13 Epidemiology Unit, Institut Pasteur de Madagascar, Antananarivo, Madagascar; 14 AP-HP, Paris Saclay, Public Health, Medical Information, Clinical Research, Le Kremlin-Bicêtre, France; Instituto de Salud Global de Barcelona, SPAIN

## Abstract

**Background:**

Severe bacterial infections (SBIs) are a leading cause of neonatal deaths in low- and middle-income countries (LMICs). However, most data came from hospitals, which do not include neonates who did not seek care or were treated outside the hospital. Studies from the community are scarce, and few among those available were conducted with high-quality microbiological techniques. The burden of SBI at the community level is therefore largely unknown. We aimed here to describe the incidence, etiology, risk factors, and antibiotic resistance profiles of community-acquired neonatal SBI in 3 LMICs.

**Methods and findings:**

The BIRDY study is a prospective multicentric community-based mother and child cohort study and was conducted in both urban and rural areas in Madagascar (2012 to 2018), Cambodia (2014 to 2018), and Senegal (2014 to 2018). All pregnant women within a geographically defined population were identified and enrolled. Their neonates were actively followed from birth to 28 days to document all episodes of SBI. A total of 3,858 pregnant women (2,273 (58.9%) in Madagascar, 814 (21.1%) in Cambodia, and 771 (20.0%) in Senegal) were enrolled in the study, and, of these, 31.2% were primigravidae. Women enrolled in the urban sites represented 39.6% (900/2,273), 45.5% (370/814), and 61.9% (477/771), and those enrolled in the rural sites represented 60.4% (1,373/2,273), 54.5% (444/814), and 38.1% (294/771) of the total in Madagascar, Cambodia, and Senegal, respectively. Among the 3,688 recruited newborns, 49.6% were male and 8.7% were low birth weight (LBW). The incidence of possible severe bacterial infection (pSBI; clinical diagnosis based on WHO guidelines of the Integrated Management of Childhood Illness) was 196.3 [95% confidence interval (CI) 176.5 to 218.2], 110.1 [88.3 to 137.3], and 78.3 [59.5 to 103] per 1,000 live births in Madagascar, Cambodia, and Senegal, respectively. The incidence of pSBI differed between urban and rural sites in all study countries. In Madagascar, we estimated an incidence of 161.0 pSBI per 1,000 live births [133.5 to 194] in the urban site and 219.0 [192.6 to 249.1] pSBI per 1,000 live births in the rural site (*p* = 0.008). In Cambodia, estimated incidences were 141.1 [105.4 to 189.0] and 85.3 [61.0 to 119.4] pSBI per 1,000 live births in urban and rural sites, respectively (*p* = 0.025), while in Senegal, we estimated 103.6 [76.0 to 141.2] pSBI and 41.5 [23.0 to 75.0] pSBI per 1,000 live births in urban and rural sites, respectively (*p* = 0.006). The incidences of culture-confirmed SBI were 15.2 [10.6 to 21.8], 6.5 [2.7 to 15.6], and 10.2 [4.8 to 21.3] per 1,000 live births in Madagascar, Cambodia, and Senegal, respectively, with no difference between urban and rural sites in each country. The great majority of early-onset infections occurred during the first 3 days of life (72.7%). The 3 main pathogens isolated were *Klebsiella* spp. (11/45, 24.4%), *Escherichia coli* (10/45, 22.2%), and *Staphylococcus* spp. (11/45, 24.4%). Among the 13 gram-positive isolates, 5 were resistant to gentamicin, and, among the 29 gram-negative isolates, 13 were resistant to gentamicin, with only 1 E. *coli* out of 10 sensitive to ampicillin. Almost one-third of the isolates were resistant to both first-line drugs recommended for the management of neonatal sepsis (ampicillin and gentamicin). Overall, 38 deaths occurred among neonates with SBI (possible and culture-confirmed SBI together). LBW and foul-smelling amniotic fluid at delivery were common risk factors for early pSBI in all 3 countries. A main limitation of the study was the lack of samples from a significant proportion of infants with pBSI including 35 neonatal deaths. Without these samples, bacterial infection and resistance profiles could not be confirmed.

**Conclusions:**

In this study, we observed a high incidence of neonatal SBI, particularly in the first 3 days of life, in the community of 3 LMICs. The current treatment for the management of neonatal infection is hindered by antimicrobial resistance. Our findings suggest that microbiological diagnosis of SBI remains a challenge in these settings and support more research on causes of neonatal death and the implementation of early interventions (e.g., follow-up of at-risk newborns during the first days of life) to decrease the burden of neonatal SBI and associated mortality and help achieve Sustainable Development Goal 3.

## Abstract 2

In a community-based, prospective cohort study, Bich-Tram Huynh and colleagues investigate the incidence and factors associated with several bacterial infections among neonates in rural and urban areas of three low-middle income countries.

## Author summary

**Why was this study done?:**

- Severe bacterial infections (SBIs) are a leading cause of neonatal deaths in low- and middle-income countries (LMICs).
- In LMICs, most data come from studies conducted in hospitals. However, a significant proportion of neonates do not have access to the hospital or die before reaching it in these countries.
- The magnitude of SBI and its causative pathogens, consequences, and risk factors at the community level remain largely unknown in LMICs.

**What did the researchers do and find?:**

- We conducted a prospective multicentric, rural and urban, community-based mother and child cohort study in Madagascar, Cambodia, and Senegal. All pregnant women within a geographically defined population were identified and invited to participate. Their neonates were actively followed from birth to 28 days.
- Among the 3,688 recruited newborns, 514 neonates presented an episode of SBI, with the great majority occurring during the first 3 days of life (54.7%). The incidence of possible severe bacterial infection (pSBI) varied from 78.3 [95% confidence interval [CI] 59.5 to 103] to 196.3 [176.5 to 218.2] cases per 1,000 live births and was the highest in Madagascar; the incidence of confirmed severe bacterial infection (cSBI) varied from 6.5 [2.7 to 15.6] to 15.2 [10.6 to 21.8] per 1,000 live births.
- The 3 main pathogens isolated were *Klebsiella* spp. (24.4%), *Escherichia coli* (22.2%), and *Staphylococcus* spp. (26.7%). Almost one-third of the isolates (31%) were resistant to both first-line drugs recommended by WHO for the management of neonatal sepsis.

**What do these findings mean?:**

- Our findings show the high burden of neonatal SBI, particularly in the first 3 days of life, in the community of 3 LMICs in Asia and Africa.
- These findings also suggest that the current treatment strategy for the management of neonatal infection may be hindered by antimicrobial resistance.
- Our findings support the implementation of early interventions (e.g., follow-up of at-risk newborns during the first days of life) to decrease the burden of severe neonatal infection and mortality and help achieve Sustainable Development Goal 3.

## Introduction

Neonatal mortality accounts for 40% of the global under-5 mortality, with the great majority occurring in low- and middle-income countries (LMICs) [1]. Severe infections are a leading cause of neonatal deaths (>75%), with an estimate of 6.9 million cases of possible severe bacterial infection (pSBI) occurring yearly in these countries [2,3].

LMICs also combine a number of risk factors for the emergence and dissemination of antibiotic-resistant bacteria (ARB) leading to potential treatment failure and increased neonatal mortality [4].

Data on the burden and risk factors of neonatal severe bacterial infections (SBIs) and ARB infections are scarce in LMICs, particularly in Africa and Asia. Data that do exist are mainly provided by hospital-based investigations, which can miss a large proportion of newborns who did not seek care or were treated outside the hospital and for which microbiological information is not available [5,6]. Only few studies have been conducted at the community level with reliable clinical and microbiology data on SBI in these countries [5,7]. These gaps in knowledge prevent the implementation of simplified treatment of SBI in the community when referral is not possible as well as the improvement of prevention and treatment interventions of neonatal SBI at the community level [8].

Investigating community-acquired neonatal SBI in LMICs is extremely challenging regarding the specificities of the context. To address these challenges, we implemented a prospective cohort study including exhaustive community recruitment and active follow-up of pregnant women and their neonates in 3 LMICs. Here, we report the incidence, etiology, risk factors, and antibiotic resistance profiles of community-acquired neonatal SBI in Madagascar, Cambodia, and Senegal.

## Methods

### Study areas and design

The full protocol is described in detail in the Supporting information (S1 Text). In Madagascar (2012 to 2018), the study was implemented in 3 urban districts of Antananarivo (population: 14,997) and in the rural city of Moramanga (population: 17,159). In Cambodia (2014 to 2018), the study was conducted in 2 areas of Steung Meanchey, an urban district of Phnom Penh (population: 87,035) and 2 districts of rural Kampong Speu (79,000 inhabitants). In Senegal (2014 to 2018), the study took place in a district of Guédiawaye, a city near urban Dakar (population: 20,529), and Sokone, a rural city close to the Gambia border (population: 14,500).

Study recruitment included the exhaustive identification of pregnant women within the study area with the help of community healthcare workers who identified pregnant women eligible for inclusion. The study area was defined based on the expected numbers of live births and a target number of inclusions. Participants were enrolled during their third trimester of pregnancy and were actively monitored to include their newborn at birth. In addition, we optimized the exhaustiveness of live birth recruitment by also including neonates at the time of birth, without a prior inclusion phase of the mother if she was not identified during her pregnancy.

Inclusion criteria for pregnant women included routine residence in the study area with no plan to move away during the follow-up period and no opposition to the research being conducted or to the collection of biological samples.

At delivery, neonates who met the following criteria were enrolled: neonates born to parents living in the study area with no plan to move during the follow-up period; those whose legal guardians were informed and had no objection to the study procedures and collection of biological samples; and those for whom written consent was obtained from at least 1 legal guardian. Data collected were the mothers’ sociodemographic, medical, and obstetric characteristics; delivery information; risk factors of infection at delivery; and the neonates’ anthropometric measurements and Apgar scores.

After delivery, we performed both active and passive surveillance during the neonatal period to detect all symptoms of severe infection [9]. Surveillance visits were scheduled for the third day after delivery and then weekly until the first month of life. Passive surveillance consisted of asking the family to contact the investigators if the newborn presented symptoms suggestive of infection. All cases of suspected infections were investigated by a physician. Biological samples (C-reactive protein and complete blood count) as well as samples for bacterial identification including blood cultures were performed according to study guidelines and the physician’s discretion. This study is reported as per the Strengthening the Reporting of Observational Studies in Epidemiology (STROBE) guideline (S1 Checklist).

### Sample size

The sample size was determined in part by available funding, which was different for each country; we aimed to enroll 750, 800, and 2,000 newborns in Senegal, Cambodia, and Madagascar, respectively.

The primary objective of the BIRDY study was to estimate the incidence of SBI at the community level. At the time of study planning, published neonatal infection incidence ranged from 6.3 to 23 per 1,000 live births, depending on the study [10–14]. In addition, WHO considered that all-cause neonatal mortality rates to be underestimated by at least 20% [15]. Given that about 36% of these deaths are attributable to severe infections, we hypothesized that this underestimation of the number of deaths probably resulted in a similar underestimation of the incidence of bacterial infections [16]. After correction for this underestimation and assuming that the number of neonates at risk remained constant over the period considered, we could expect, with the targeted recruitment number, maximal incidences, by site, of between 8 per thousand (95% confidence interval (CI) [2.5 to 13.5]) and 28 per thousand (95% CI [17.6 to 37.4]) per 1,000 live births.

### Bacteriologic analyses

All samples were immediately stored at a temperature between 0 and 4°C and transported within hours to the clinical laboratory at the Institut Pasteur of each country. All analyses were performed according to the guidelines from the French Society for Microbiology (S1 Text) [17]. For blood culture, fresh blood agar and chocolate media were used for bacterial isolation, and bacterial strains from the cultures were identified using API Gallery or matrix-assisted laser desorption/ionization time-of-flight (MALDI-TOF) mass spectrometry. Antimicrobial susceptibility testing was done by disk diffusion in accordance with the French Society for Microbiology guidelines [17]. A double-disk synergy test was systematically done in all Enterobacteriaciae resistant to third-generation cephalosporin to identify extended-spectrum β-lactamase (ESBL) producers. *Escherichia coli* ATCC 25922 was used for quality control strains.

### Case ascertainment

The neonatal period was defined from birth to 28 days (i.e., day 0 to 28) of life.

We defined pSBI cases as the presence of at least 1 sign among severe chest indrawing, tachypnea, hypo- or hyperthermia, lethargy, convulsions and poor feeding, and no pSBI nor hospitalization within the previous 7 days. Neonates presenting tachypnea as the only clinical sign were not counted as cases because of the low specificity of this sign alone [7].

We defined confirmed severe bacterial infection (cSBI) as presence of at least 1 sign cited above and a positive culture from blood or cerebrospinal fluid (CSF) or urine (bacterial and leukocyte counts >10^5^ and 10^4^, respectively) or pus from umbilicus/subcutaneous abscess in case of soft tissue or omphalitis-associated sepsis.

All cases of infection were reviewed by an epidemiologist (BTH), a neonatologist (EKD), and a microbiologist (BG or JMC) to classify them as cSBI or pSBI and to exclude nonsevere cases or clinically nonsignificant contaminant.

Early-onset infections were defined as cases occurring between 0 and 6 days and late-onset infections as cases occurring between 7 and 28 days. The time when day 0 became day 1 was set at midnight.

Multidrug-resistant infections were those caused by pathogens resistant to >1 agent in >3 antibacterial categories [18].

### Statistical analyses

Incidence estimation was planned a priori. The analysis plan for risk factors determination of SBI was developed prospectively, with later adjustments, such as running a Cox model with a stratification for covariate violating the proportional hazards assumption according to reviewers’ suggestions, and no data-driven changes took place to the analysis plan.

Incidences rates of cSBI and pSBI were expressed as neonatal infections per 1,000 live births.

Maternal characteristics and obstetrical and neonatal outcomes were compared between countries by the use of Fisher exact or χ^2^ tests for proportions and *t* tests for mean values. Very low birth weight (VLBW) and low birth weight (LBW) were defined as a birth weight of less than 1,500 g and less than 2,500 g, regardless of gestational age. Dystocic delivery is defined as abnormal labor resulting from abnormalities of the power (uterine contractions or maternal expulsive forces), the passenger (position, size, or presentation of the fetus), or the passage (pelvis or soft tissues).

We investigated in each country if LBW and factors related to delivery (foul-smelling amniotic fluid, premature rupture of membranes, dystocic delivery, and cesarean section) (exposures of primary interest) were associated with early pSBI, as it represented the majority of the overall burden and considered site, sex of the newborns, and maternal and pregnancy characteristics as potential confounding factors. We first conducted a univariate analysis to describe the relationship between the outcome and the exposures of primary interest and potential cofounding factors. Then, we performed a multivariable analysis to present adjusted analysis for the exposures of interest. In univariate analysis, the associations with risk of early pSBI were estimated using Cox proportional hazards models and expressed as hazard ratios (HRs) with 95% CI. The proportional hazards assumption was tested using Schoenfeld residuals for each independent variable and outcome pair.

For multivariable analysis, factors associated with the outcome were entered in the initial model if *p*-value was less than 0.20 in univariate analysis. A stepwise backward procedure was then performed to identify factors independently associated with the outcome (*p* < 0.05). If a variable violated the proportional hazards assumption, this variable was considered as a stratification factor in the Cox proportional hazard model. To document the direction and the strength of the variable that violated the proportional hazards assumption, an additional parametric regression model with an accelerated failure time approach and a log-normal distribution was used.

Two variables were highly correlated: foul-smelling amniotic fluid and premature rupture of the membranes; we therefore kept the variable that was the most associated with the considered outcome (foul-smelling amniotic fluid) to avoid multicollinearity in multivariable analysis.

In Senegal, 14% of neonates had missing values for variables related to risk factors at delivery (foul-smelling amniotic fluid, premature rupture of membranes, and dystocic delivery), mainly for logistic reasons. Consequently, missing data were classified as missing completely at random (MCAR). We performed complete case analyses that provide unbiased estimates under MCAR assumption and also made sensitivity analyses incorporating worst- and best-case scenarios [19,20]. In the worst scenario, infants were classified as infants born to mothers with foul-smelling amniotic fluid and/or premature rupture of membranes and/or dystocic delivery depending on which variable was missing. In the best scenario, infants were classified as infants as infants born to mother with no foul-smelling amniotic fluid and/or no premature rupture of membranes and/or who had a eutocic delivery.

All analyses were performed using Stata version 15 (Stata, College Station, Texas, United States of Americs). All *p*-values were from 2-sided tests, and results were deemed statistically significant at *p* < 0.05.

### Ethical statement

The study was approved by the ethics committees of Madagascar (068-MSANP/CE), Senegal (SEN 14–20), Cambodia (108 NEHCR), and the Institutional Review Board of Institut Pasteur (IRB/2016/08/03), France. Written informed consent was given by all participants.

## Results

**Fig 1:**
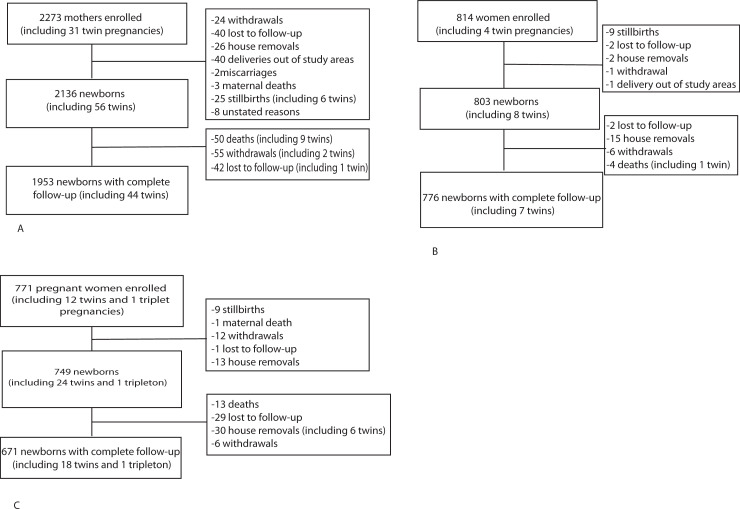
Flowchart of the study population. **(A)** Madagascar, **(B)** Cambodia, and **(C)** Senegal.

A total of 3,858 pregnant women were enrolled in the study: 2,273 (58.9%) in Madagascar, 814 (21.1%) in Cambodia, and 771 (20%) in Senegal (Fig 1). Women enrolled in the urban sites represented 39.6%, 45.5%, and 61.9% of the total in Madagascar, Cambodia, and Senegal, respectively. Maternal and neonatal characteristics by country are presented in Table 1. A total of 3,688 (95.6%) babies were born in the frame of the study. Among the 3,688 recruited newborns, 49.6% were male, and 75.6% were born in a healthcare facility. There were 10.6% (221/2,078), 4.1% (33/792), and 9.6% (68/705) LBW in Madagascar, Cambodia, and Senegal, respectively. Among them, 50% (108/216), 39.4% (13/33), and 12.7% (7/55) were born prematurely (before 37 weeks gestation) in Madagascar, Cambodia, and Senegal, respectively. There were 73 infants (2%) lost to follow-up (42 in Madagascar, 2 in Cambodia, and 29 in Senegal). In total, 3,414 (92.6%) newborns (1,953 in Madagascar, 776 in Cambodia, and 671 in Senegal) had a complete follow-up during the neonatal period.

**Table 1:** Characteristics of the mothers and their neonates.

	Madagascar	Cambodia	Senegal	*P*
**Mothers**	***N* = 2,273**	***N* = 814**	***N* = 771**	
**Urban site**	39.6 (900/2,273)	45.5 (370/814)	62.6 (477/762)	<0.001
**Age, median (SD) [range]**	25 (6.5) [14–48]	27.0 (5.4) [14–46]	27.9 (6.5) [15–49]	0.20
**Education**				
Absence/primary school	24.8 (564/2,273)	54.7 (445/814)	73.5 (560/762)	<0.001
Partial secondary school	52.3 (1,188/2,273)	31.8 (259/814)	18.0 (137/762)	
Complete secondary or higher	22.9 (521/2,273)	13.5 (110/814)	8.5 (65/762)	
**Electricity**	75.4 (1,714/2,273)	99.5 (809/813)	97.1 (740/762)	<0.001
**Latrine**				
Inside the house	7.3 (166/2,273)	50.3 (409/813)	51.1 (387/757)	<0.001
Outside the house	92.7 (2,106/2,273)	49.7 (405/813)	48.9 (370/757)	
**Household members median [range]**	4 [2–14]	5 [2–21]	10 [2–21]	0.03
**Primigravidae**	32.8 (746/2,273)	33.8 (275/814)	23.5 (179/762)	<0.001
**Twin pregnancy**	1.4 (31/2,273)	0.4 (4/814)	1.6 (13/771)	0.07
**At least 1 antenatal visit at enrollment**	97.6 (2,218/2,273)	98.6 (761/772)	99.3 (722/727)	<0.001
**Skilled birth attendant**	72.2 (1,528/2,117)	99.4 (787/791)	95.3 (726/762)	<0.001
**Hospitalization during pregnancy**	2.6 (54/2,098)	3.2 (26/812)	3.0 (21/687)	0.45
**Babies**	***N* = 2,136**	***N* = 803**	***N* = 749**	
**Male**	50.7 (1,080/2,128)	48.0 (381/794)	50.2 (376/749)	0.29
**Birth weight, median (SD) [range]**	2,969.1 (469) [1,000–4,900]	3,104.1 (437.3) [1,500–4,600]	3,049.6 (497) [1,000–5,000]	0.10
**LBW** ^**a**^	10.6 (221/2,078)	4.1 (33/792)	9.6 (68/705)	<0.001
**VLBW** ^**b**^	0.7 (15/2,078)	0 (0/792)	0.2 (2/705)	0.02
**Preterm** ^**c**^	15.5 (329/2,121)	2.2 (17/777)	4.5 (31/689)	<0.001
**Delivery in healthcare facilities**	60.2 (1,276/2,119)	98.6 (782/793)	98.5 (733/744)	<0.001
**Cesarean section**	9.3 (198/2,135)	12.5 (99/794)	3.4 (25/739)	<0.001
**Premature rupture of the membranes**	2.5 (54/2,120)	0.5 (4/769)	4.3 (27/630)^d^	<0.001
**Fetid amniotic fluid**	7.8 (166/2,119)	2.1 (16/769)	18.3 (115/630)^d^	<0.001
**Dystocic delivery**	8.5 (181/2,119)	4.6 (35/769)	2.2 (14/630)^d^	<0.001
**Exclusive breastfeeding at day 7**	89 (1,826/2,051)	77.1 (586/760)	94.8 (688/726)	0.03

^a^ LBW is defined as a birth weight<2,500 g.

^b^ VLBW is defined as a birth weight<1,500 g.

^c^ Birth before 37 weeks gestation.

^d^ In Senegal, data related to delivery (premature rupture of the membranes, fetid amniotic fluid, and dystocic delivery) are for 630 neonates among 749 due to missing data.

Data are % (*n*/*N*) unless stated otherwise.

LBW, low birth weight; VLBW, very low birth weight.

### Samples

**Fig 2:**
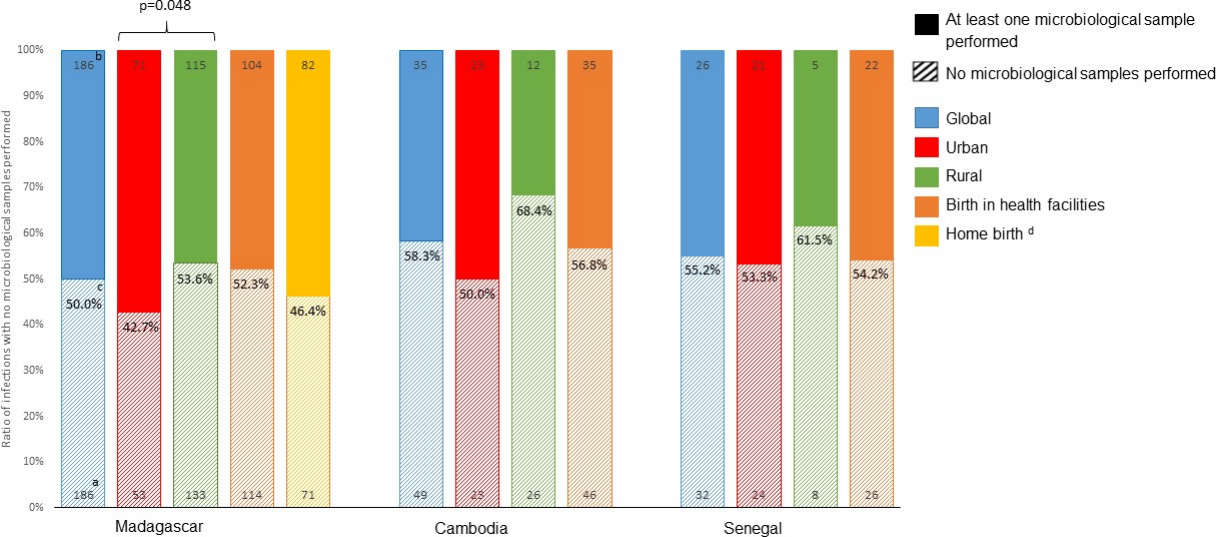
Ratio of SBIs without microbiological samples performed. Total SBIs include both culture-confirmed SBI and pSBI. **(a)** Numbers at the bottom of each bar represent the number of neonatal SBI with no microbiological samples performed. **(b)** Numbers at the top of each bar represent the number of neonatal SBI with at least 1 microbiological sample performed among blood culture, urine test, and lumbar puncture. **(c)** Percentages in the middle of each bar represent the ratio of neonatal SBI with no microbiological samples performed (number of neonatal SBI without microbiological samples performed/ overall number of neonatal SBI). **(d)** Only in Madagascar (>98% of the pregnant women delivered in healthcare facilities in Cambodia and Senegal). pSBI, possible severe bacterial infection; SBI, severe bacterial infection.

A total of 514 cases of SBI (42 culture confirmed and 472 pSBI) were identified. Among these, 247 had at least 1 microbiological sample (215 blood cultures, 101 urine tests, and 26 lumbar punctures). The proportion of cases with no microbiological sample taken was 50% (186/372), 58.3% (49/84), and 55.2% (32/58) in Madagascar, Cambodia, and Senegal, respectively (Fig 2). The proportion of unexplored pSBI was high in the rural site in the 3 countries (53.6%, 68.4%, and 61.5% in Madagascar, Cambodia, and Senegal, respectively), although significantly different only in Madagascar when compared to the urban site (*p* = 0.048). Among SBI cases, those who experienced dystocic delivery were less likely to have a microbiological sample taken compared to those born without delivery complication (60.56% versus 47.64% (*p* = 0.05)) in Madagascar. Also, neonates with SBI who needed resuscitation at birth were less likely to be sampled compared to those who did not need resuscitation in Senegal (85.7% versus 47.9% (*p* = 0.06)), although this difference was not statistically significant.

### Incidence of severe bacterial neonatal infections

#### pSBIs

The overall incidence of pSBI was 196.3 per 1,000 live births [95% CI = 176.5 to 218.2], 110.1 [88.3 to 137.3], and 78.3 [59.5 to 103] in Madagascar, Cambodia, and Senegal, respectively.

The incidence of pSBI differed between urban and rural sites in all study countries. In Madagascar, we estimated an incidence of 161.0 pSBI per 1,000 live births [133.5 to 194.0] in the urban site and 219.0 [192.6 to 249.1] pSBI per 1,000 live births in the rural site (*p* = 0.008). In Cambodia, estimated incidences were 141.1 [105.4 to 189.0] and 85.3 [61.0 to 119.4] pSBI per 1,000 live births in urban and rural sites, respectively (*p* = 0.025), while in Senegal, we estimated 103.6 [76.0 to 141.2] pSBI and 41.5 [23.0 to 75.0] pSBI per 1,000 live births in urban and rural sites, respectively (*p* = 0.006).

**Fig 3:**
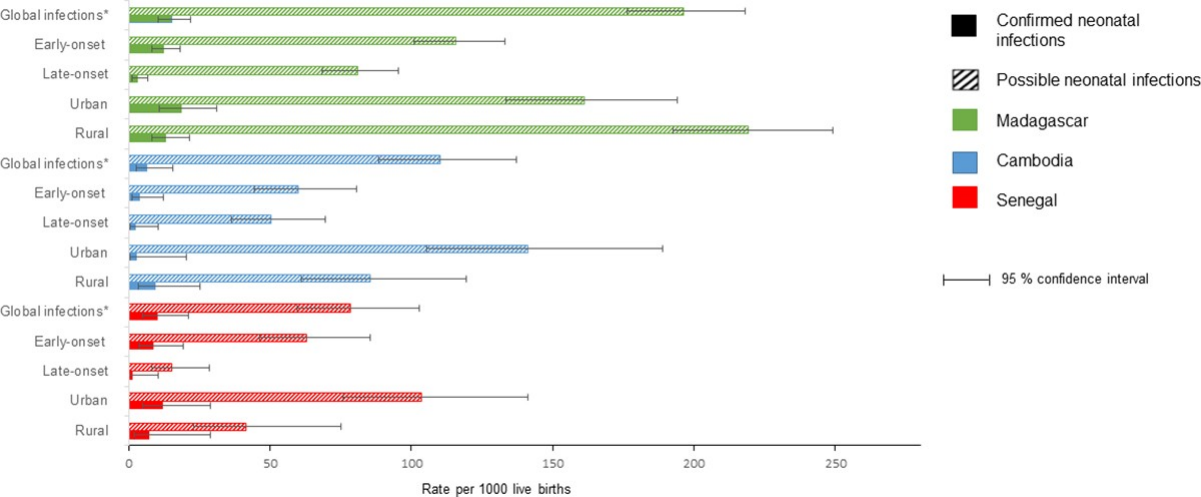
Incidence of severe neonatal infections (per 1,000 live births). Early-onset infections are defined as cases occurring between 0 and 6 days and late-onset infections as cases occurring between 7 and 28 days.* Global infections incidence is defined as early-onset and late-onset infections together.

The early pSBI incidences were estimated at 115.9 per 1,000 live births [101 to 133], 59.9 [44.4 to 80.8], and 63 [46.3 to 85.5] in Madagascar, Cambodia, and Senegal, respectively. The incidence of late pSBI ranged from 15.4 [8.3 to 28.5] in Senegal to 80.9 [68.6 to 95.4] per 1,000 live births in Madagascar (Fig 3, S1 Table).

#### cSBIs

A total of 42 neonates were classified as having culture-confirmed severe infection (*n* = 30 in Madagascar, *n* = 5 in Cambodia, and *n* = 7 in Senegal). The overall incidence rates of cSBI per 1,000 live births were 15.2 [10.6 to 21.8], 6.5 [2.7 to 15.6], and 10.2 [4.8 to 21.3] in Madagascar, Cambodia, and Senegal, respectively, with no difference between the 3 countries and between urban and rural sites in each country (S1 Table).

The early-onset severe neonatal infections rates ranged from 3.9 [1.3 to 12.1] in Cambodia to 12.3 [8.2 to 18.3] per 1,000 live births in Madagascar, and late-onset infection rates ranged from 1.4 [0.2 to 10.3] in Senegal to 3.1 [1.4 to 6.8] per 1,000 live births in Madagascar.

The great majority of early-onset infections occurred during the first 3 days of life (16/24 in Madagascar, 3/3 in Cambodia, and 5/6 in Senegal).

**Fig 4:**
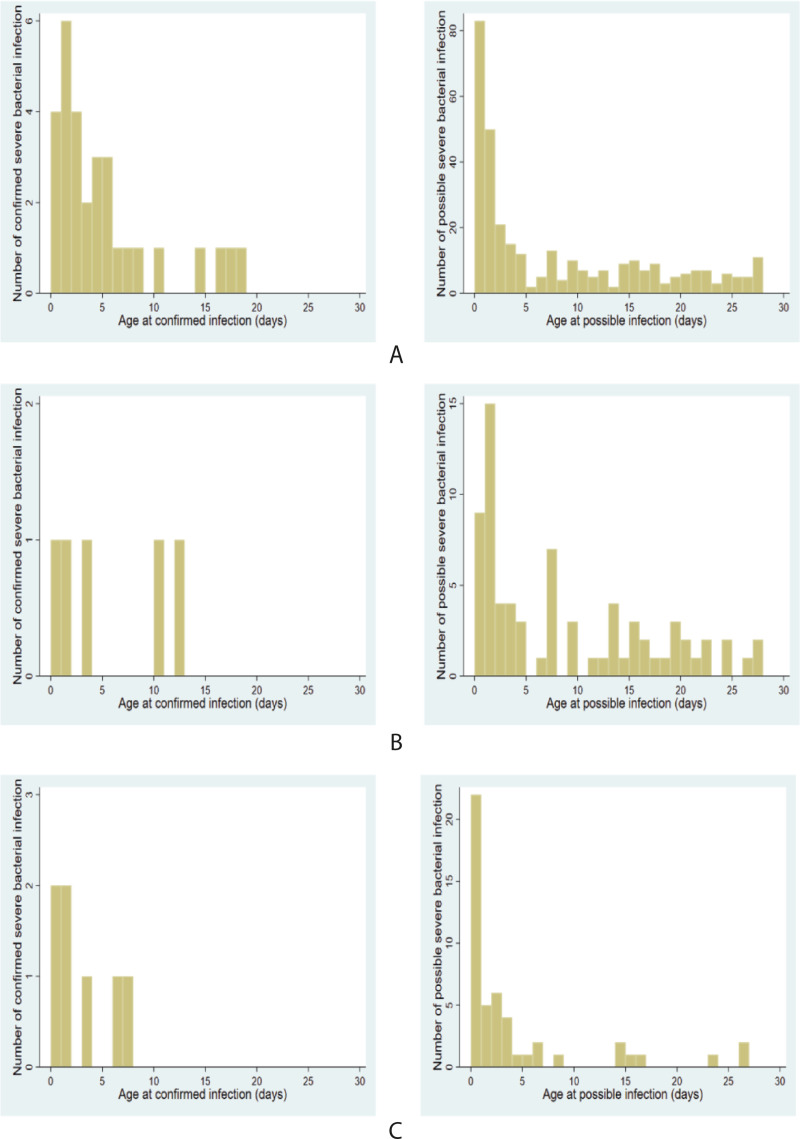
Timing of cSBI and pSBI among neonates. **(A)** Madagascar, **(B)** Cambodia, and **(C)** Senegal. cSBI, confirmed severe bacterial infection; pSBI, possible severe bacterial infection.

In the 3 countries, all estimates of pSBI incidences largely surpassed those of cSBI: 12.9, 16.9, and 7.7 times greater in Madagascar, Cambodia, and Senegal, respectively. Among the 514 episodes of SBI, 54.7% occurred during the first 3 days (Fig 4).

### Pathogens isolated and antibacterial resistance

#### Pathogens

Among the 42 neonates with cSBI, 45 pathogens were identified including 3 samples in which 2 pathogens were isolated (S2 Table). The 3 main pathogens isolated were *Klebsiella* spp. (11/45, 24.4%) *E*. *coli* (10/45, 22.2%), and *Staphylococcus* spp. (11/45, 24.4%).

Overall, we identified 5 cases of meningitis and 37 cases of bacteremia/sepsis including 7 secondary to urinary tract infection, 5 secondary to omphalitis, and 1 secondary to a muscular abscess.

#### Antibacterial resistance

Among the 45 bacteria isolated, 42 were tested for antimicrobial drug susceptibility (29/32 gram-negative and 13/13 gram-positive isolates) (S3 Table). More than half of isolates with resistance data (23/42, 54.8%) were resistant to at least 1 of the first-line drugs recommended for the management of neonatal sepsis (ampicillin and gentamicin), with 13/42 (31%) resistant to both drugs (8 *Klebsiella* spp., 4 *E*. *coli*, and 1 *Enterobacter cloacae*).

Overall, 18 isolates were identified as multidrug resistant (44.8% (13/29), 20% (1/5), and 50% (4/8) in Madagascar, Cambodia, and Senegal, respectively), which led to an estimation of the overall incidence rate for multidrug-resistant severe neonatal infection of 5.0 [3.1 to 8.0] cases per 1,000 live births.

Almost half of the gram-negative isolates were resistant to cefotaxime (48%, 12/25) and to gentamicin (46.4%, 13/28), and more than one-third showed resistance to ciprofloxacin (34.5%, 10/29). No resistance was detected toward imipenem. Moreover, 11 Enterobacteriaceae were ESBL producers: 8 in Madagascar and 3 in Cambodia.

Among the 5 *Staphylococcus aureus* and the 5 coagulase-negative staphylococci (CoNS) isolated, 1 and 2 strains were resistant to methicillin, respectively.

### Clinical presentation and outcomes

Among the 472 neonates with pSBI, the most prevalent clinical signs were fever (51%), feeding difficulties (28.2%), lethargy (17.5%), irritability (12.7%), and difficult breathing (13.5%). For neonates presenting cSBI, fever (61.9%), feeding difficulties (31.8%), irritability (31.8%), lethargy (22.7%), difficult breathing (15.9%), prolonged capillary refill (>3 seconds) (11.9%), and cyanosis (9.1%) were the most frequent clinical signs.

There were 3 deaths among neonates with cSBI caused by *E*. *cloacae* at day 15 in Senegal, *Klebsiella pneumoniae* at day 5, and *Acinetobacter baumannii* at day 20 in Madagascar. All 3 deaths occurred among hospital births and were due to resistant bacteria that required imipenem.

Among neonates with pSBI, 26, 2, and 7 deaths occurred in Madagascar, Cambodia, and Senegal, respectively. Among these neonates, infection fatality ratio of early-onset infection was 11.4% (23/201), [CI = 7.4 to 16.7%] in Madagascar, 4.7% (2/43), [CI = 0.6 to 15.8%] in Cambodia, and 4.9% (2/41), [CI = 0.6 to 16.5%] in Senegal, with no statistically significant difference between the 3 countries (*p* = 0.22). Infection fatality ratio of late-onset infection was 2.1% (3/141), [CI = 0.4 to 6%] in Madagascar and 50% (5/10), [CI = 18.7 to 81.3%] in Senegal; no death occurred among late-onset pSBI in Cambodia.

### Factors associated with early pSBI

We found in all 3 countries that neonates with foul-smelling amniotic fluid at delivery (adjusted hazard ratio (aHR) = 2.2 [1.71 to 3.4], *p* < 0.001 in Madagascar, aHR = 3.94 [2.05 to 7.59], *p* < 0.001 in Senegal, and 5.5 [2.1 to 14.4], *p* = 0.001 in Cambodia) presented a higher risk of early pSBi (Table 2). Also, LBW babies (aHR = 4.52; 95% CI [3.36 to 6.1], *p* < 0.001 in Madagascar, aHR = 2.48 [1.03 to 5.95], *p* = 0.04 in Senegal were at increased risk of early pSBi compared to normal weight babies (Table 2). In Cambodia, the variable “LBW” violated the proportional hazards assumption and was considered as a stratification variable. To document the direction and the strength of “LBW” in Cambodia, a parametric regression model with an accelerated failure time approach and a log-normal distribution were used (S4 Table). In the adjusted analysis, in neonates with LBW, time to onset of SBI was about 10 times shorter than in neonates with normal birth weight (adjusted time ratio (aTR): 0.11 [0.05 to 0.24], *p* < 0.001).

**Table 2:** Risk factors analysis of possible severe bacterial neonatal infections.

	Madagascar *N* = 2,050				Senegal *N* = 630				Cambodia^d^ *N* = 769			
	Univariate analysis		Multivariable analysis		Univariate analysis		Multivariable analysis		Univariate analysis		Multivariable analysis	
	HR [95% CI]	*p*	Adjusted HR^a^ [95% CI]	*p*	HR [95% CI]	*p*	aHR^b^ [95% CI]	*p*	HR [95% CI]	*p*	aHR^c^ [95% CI]	*p*
**Site (urban site as reference)**	0.9 [0.75–1.3]	0.88			0.58 [0.29–1.14]	0.12			0.8 [0.46–1.45]	0.48		
**Education**												
Absence/primary school	Ref				Ref				Ref			
Partial secondary school	0.81 [0.58–1.11]	0.19			0.82 [0.31–2.12]	0.68			0.83 [0.44–1.58]	0.57		
Complete secondary or higher	1.1 [0.77–1.58]	0.59			1.44 [0.56–3.74]	0.45			0.55 [0.19–1.56]	0.26		
**Primigravidae**	1.36 [1.03–1.79]	0.03			0.94 [0.43–2.04]	0.87			1.5 [0.86–2.76]	0.14		
**Twins pregnancy**	3.99 [2.5–6.4]	0.001			- ^e^				7.03 [1.7–29]	0.007		
**Hospitalization during pregnancy**	2.02 [1.1–3.7]	0.02			0.87 [0.12–6.3]	0.89			0.7 [0.1–5.1]	0.72		
**Skilled birth attendant**	0.95 [0.7–1.31]	0.77			0.6 [0.08–4.3]	0.61			- ^f^			
**Sex of newborn (boys as reference)**	0.72 [0.55–0.94]	0.02			0.9 [0.5–1.7]	0.80			0.8 [0.45–1.4]	0.45		
**Delivery out of healthcare facilities**	0.8 [0.6–1.04]	0.10			- ^e^				- ^f^			
**LBW**	4.35 [3.28–5.8]	0.001	4.52 [3.36–6.1]	<0.001	2.39 [1.0–5.7]	0.05	2.48 [1.03–5.95]	0.04	- ^**d**^			
**Cesarean section**	1.65 [1.14–2.4]	0.001			1.7 [0.23–12.4]	0.53			1.34 [0.6–3]	0.48		
**Fetid amniotic fluid**	3.2 [2.3–4.44]	0.001	2.2 [1.71–3.4]	<0.001	3.78 [1.99–7.16]	0.001	3.94 [2.05–7.6]	<0.001	6.01 [2.38–15.2]	<0.001	5.5 [2.1–14.4]	0.001
**Dystocic delivery**	4.15 [3.08–5.58]	0.001	3.2 [2.38–4.5]	<0.001	2.8 [0.67–11.6]	0.16			2.2 [0.77–6.04]	0.14		

^a^ Multivariable analysis in Madagascar: adjusted on site (rural versus urban) (1.2 [0.88–1.55], *p* = 0.28) and sex of the newborn (0.65 [0.49–0.86], *p* = 0.002).

^b^ Multivariable analysis in Senegal: adjusted on site (rural versus urban) (0.51 [0.25–1.01], *p* = 0.05) and sex of the newborn (1.06 [0.55–2.01], *p* = 0.87).

^c^ Multivariable analysis in Cambodia: adjusted on site (rural versus urban) (0.91 [0.5–1.65], *p* = 0.75) and sex of the newborn (0.7 [0.38–1.26], *p* = 0.23).

^d^ For analysis in Cambodia: LBW is incorporated in the model as a stratification factor.

^e^ No possible estimation as no cases of pSBI occurred among twins and one case among neonates who delivered out of healthcare facilities.

^f^ No possible estimation as no cases of pSBI occurred among neonates who delivered out of healthcare facilities and among those who delivered with unqualified healthcare workers.

aHR, adjusted hazard ratio; CI, confidence interval; HR, hazard ratio; LBW, low birth weight; pSBI, possible severe bacterial infection.

Complete case analyses and sensitivity analyses (worst- and best-case scenarios) yielded comparable results except for LBW, which was not significantly associated with early pSBI in the worst-case scenario in Senegal (S5 Table).

## Discussion

Our results showed high incidences of SBI among neonates in 3 different LMICs in Asia and Africa using a prospective community-based approach. A great majority of SBI occurred during the first 3 days of life and rates of antibiotic resistance to recommended first-line treatments were also high, raising questions about current recommendations for the management of neonatal sepsis (ampicillin and gentamicin).

We observed a relatively high estimation of pSBI incidence, up to more than 200 cases per 1,000 live births. A recent community-based cohort with a similar design was conducted in Pakistan, India, and Bangladesh from 2011 to 2014 by Saha and colleagues [7]. However, the authors included cases of severe infections occurring until 59 days of life, whereas we considered cases during the first 28 days of life [21]. The authors found an estimation of pSBI ranging from 78.3 to 112.6 episodes per 1,000 live births. If we take into account the different follow-up time between the 2 studies, our estimation of pSBI incidence is higher, particularly in Madagascar.

Saha and colleagues performed extensive molecular diagnosis and could attribute a cause in only 28% of the episodes (16% bacterial and 12% viral). It is therefore also likely that in our study a significant proportion of the episodes were not of bacterial origin or of infectious origin as signs and symptoms of severe infection in neonates are subtle and nonspecific with some clinical signs of severe infection overlapping with noninfectious severe events (e.g., intrapartum related complication, prematurity …) [22].

Our estimates of cSBI, ranging from 6.5 to 15.2 cases per 1,000 live births, is 6 to 15 times higher than in high-income countries (<1 per 1,000 live births in the USA) [23]. Although the definition of the neonatal period differed between the 2 studies, our results are in line with estimates of Saha and colleagues, which ranged from 10.61 to 23.46 cases per 1,000 live births.

Taking into account the challenges of addressing this research question in LMICs, our methodology provided reliable estimates of both pSBI and cSBI, with the true incidence of SBI probably falling between these two. These figures underline the high burden of SBI in settings where SBI management is challenged by limited provision of basic supportive care and respiratory support and the lack of skilled staff.

Our findings also suggest that the first week and especially the first 3 days of life are critical with the majority of infections occurring during that period [7,24]. In South Asia, Saha and colleagues showed that incidence of pSBI was higher in the first 3 days with an increase in incidence density rate of roughly 13.5 times. Thus, this finding reinforces the need for interventions targeting the perinatal period.

In concordance with previous studies conducted in LMICs, we found that the most predominant bacteria responsible for neonatal infections were *Klebsiella* spp., *E*. *coli*, and *Staphylococcus* spp. [7,11,13,14,25–27]. Among the latter, a significant proportion of CoNS were identified in our study as a causative organism for early-onset infections with strains sensitive to vancomycin and teicoplanin unlike high-income countries where CoNs are responsible for late-onset sepsis acquired in hospital settings and resistant to vancomycin and teicoplanin. Our results suggest that neonates might also get infected due to unhygienic conditions during labor or nonoptimal cord care and not solely in nosocomial environments. These findings reinforce the importance of hygienic measures during delivery and for perinatal care. In Madagascar, particularly, where one-third of the pregnant women delivered at home, the use of clean birth kits should be encouraged. We did not find as Saha and colleagues that *Ureaplasma* spp. was a predominant causative bacteria as we did not culture them. Indeed, *Ureaplasma* spp. do not grow on standard media. Also, we did not perform molecular diagnosis. Further investigation including systematic testing of *Ureaplasma* spp. in neonates with pSBI are needed to confirm its burden compared to the others bacteria.

We did not identify any group B streptococcus (GBS) culture-confirmed infections, although our methodology maximized the chances to document such infections, which often occur at the early stage of the neonatal period. GBS infection might have occurred among the 35 deaths with pSBI, since only 50% of neonates with pSBI underwent microbiological investigation. Indeed, Saha and colleagues showed that the attributed proportion of GBS for pSBI was higher among babies who died than among survivors. Also, Taylor and colleagues recently conducted a study in 7 African countries aiming at determining causes of under-5 mortality with minimally invasive tissue sampling (Child Health and Mortality Prevention Surveillance (CHAMPS) study) [27]. This innovative methodology allowed the authors to capture blood samples after early death, and their preliminary results showed that GBS was a major causative bacteria among neonates with sepsis who died, along with *A*. *baumannii* (probably mostly hospital acquired), *K*. *pneumoniae*, and *E*. *coli*. Our findings highlight the challenge in estimating the burden of GBS as infected neonates might deteriorate very quickly in LMIC settings. This question is all the more important to investigate as a vaccine for GBS carriage is currently under development.

One important result of our study is that almost one-third of the overall isolates were resistant to both drugs recommended by WHO to manage neonatal sepsis (ampicillin and gentamicin). All these infections were caused by gram-negative bacilli, particularly *Klebsiella* spp., and almost half of the isolates also showed resistance to third-generation cephalosporins. No carbapenem resistance was identified.

Susceptibility of causative bacteria to drugs recommended by WHO is unclear with some studies showing results consistent with ours, whereas other show moderate resistance [7,11,27,28]. These findings suggest that the situation might be heterogeneous depending on the country, and revised recommendations should be adapted locally. Importantly, we found a relatively low incidence of multidrug-resistant infections (5 cases/1,000 live births) and did not identify carbapenemase-producing *Enterobacteriaceae*. These results suggest that antibiotic resistance to last-resort drugs is still limited in community settings in the study areas of these 3 countries, and it is still time for action to contain the spread of these strains.

One striking result of the study is the gap between pSBI and cSBI incidences, pSBI being 8 to 17 times more frequent than cSBI. This gap illustrates the challenge of microbiological diagnosis in LMIC settings. Indeed, despite a prospective design and freely available access to biological analyses, less than 50% of neonates with pSBI underwent microbiological investigation, which was at the attending clinician’s discretion. Our results suggest that microbiological diagnosis is largely underused, particularly in rural areas and most likely in critically ill babies. Specifically, we observed that lumbar puncture was not widely practiced routinely, although the protocol specified indications of CSF sampling and despite extensive refresh trainings. These findings are worrisome for optimal management of severe neonatal infection in LMICs and strengthen the pressing need for the development of innovative rapid diagnostic tests easily usable at bedside in order to identify causative pathogens and associated susceptibility pattern. Also, this bottleneck is essential to overcome in order to provide high-quality data on cause and antimicrobial resistance to meet research and surveillance needs.

LBW and foul-smelling amniotic fluid at delivery were common risk factors for early pSBI in all 3 countries. LBW can be either due to intrauterine growth retardation or prematurity. The latter has been shown to increase the risk of developing a SBI due to immaturity of the immune system and of the skin and intestine epithelial barriers [28–30]. Foul-smelling amniotic fluid often reflects ascending maternal infection from colonizing pathogens of perineum or genitourinary tract, which might be transmitted to the neonates perinatally. This result is consistent with previous studies conducted in others LMICs [31]. These findings are important as these 2 clinical signs are easily detectable by nonskilled healthcare workers, and their surveillance could be part of an intervention to identify at-risk newborns during the perinatal period, especially in settings where access to health facilities is limited.

Our study draws its strength from its rigorous methodology designed for exhaustive identification of neonatal SBI in a geographic area along with appropriate and timely diagnosis to overcome challenges in investigating community-acquired neonatal SBI in 3 LMICs of Asia and Africa.

However, we acknowledge some limitations. First, despite intensive efforts, samples could not be collected from the 35 babies with pSBI who died, most of the time because the clinical situation was too critical to sample them. Therefore, we cannot confirm that these babies died from a bacterial infection. Moreover, more than half of these newborns were LBW, indicating that others factors, such as prematurity, may have contributed to these deaths. It is also likely that mortality due to SBI in the community is higher in reality than in research settings where facilitated access to healthcare facilities, adequate treatment, and active surveillance may impact outcomes. In this context, innovative approaches such as in the CHAMPS study are important to decipher causes of mortality [27].

Second, blood cultures in neonates are particularly challenging to draw, requiring adequate blood volume in aseptic conditions. In rural areas, particularly in Senegal (6 hours), transportation time of the samples to the laboratory might have reduced the bacterial yield, resulting in negative results. Although continuous trainings to ensure blood sampling quality were delivered throughout the study, including recommendations on adequate blood volume and aseptic sampling techniques, the incidence rate of cSBI may be underestimated in our study, as already mentioned above. Also, we cannot exclude that some gut pathogens on the baby’s skin might have contaminated the blood cultures.

Finally, our findings might not be generalizable to others areas within the same country or in another LMIC. However, given the challenges in investigating neonatal sepsis at the community level in LMICs, our findings add important values to the scientific community and to policymakers of each country where data on neonatal sepsis are scarce.

This study has important policy and research implications. Our findings show the high burden of neonatal SBI in the community of 3 LMICs in Asia and Africa and also suggests that the current treatment strategy for the management of neonatal infection could be hindered by antimicrobial resistance. Our study shows that SBI is a critical issue but that even in a research setting, microbiological diagnosis of SBI remains a challenge in LMICs, impeding not only neonatal SBI management but also research and resistance surveillance. Also, our findings shows that the first 3 days are particularly vulnerable and support the implementation of early interventions (e.g., follow-up of at-risk newborns during the first days of life).

## Conclusions

The BIRDY study uses an innovative community-based approach to inform knowledge on the burden of neonatal SBI in 3 LMICs in Asia and Africa. Results from this study will help to improve prevention and treatment interventions at the community level, to decrease neonatal severe infection and associated mortality, and to help achieve Sustainable Development Goal 3.

## Supporting information

S1 Checklist STROBE checklist. STROBE, Strengthening the Reporting of Observational Studies in Epidemiology.
(DOC)

S1 Text Study protocol. (DOCX)

S1 Table Global (both early- and late-onset infections), early, and late incidences of culture-confirmed and possible severe neonatal infections according to site. (DOCX)

S2 Table Pathogens isolated in neonatal culture-confirmed severe infections. (DOCX)

S3 Table Susceptibility pattern of isolated bacteria in culture-confirmed neonatal infections. (DOCX)

S4 Table Risk factors analysis of possible severe bacterial neonatal infection in Cambodia with parametric regression model, with an accelerated failure time approach. (DOCX)

S5 Table Sensitivity analysis: risk factors analysis of pSBI in Senegal. pSBI, possible severe bacterial infection.
(DOCX)

## Abbreviations

aHR: adjusted hazard ratio
ARB: antibiotic-resistant bacteria
aTR: adjusted time ratio
CHAMPS: Child Health and Mortality Prevention Surveillance
CI: confidence interval
CoNS: coagulase-negative staphylococci
cSBI: confirmed severe bacterial infection
CSF: cerebrospinal fluid
ESBL: extended-spectrum β-lactamase
GBS: group B streptococcus
HR: hazard ratio
LBW: low birth weight
LMIC: low- and middle-income country
MALDI-TOF: matrix-assisted laser desorption/ionization time-of-flight
MCAR: missing completely at random
pSBI: possible severe bacterial infection
SBI: severe bacterial infection
STROBE: Strengthening the Reporting of Observational Studies in Epidemiology
VLBW: very low birth weight
