# Supplementary material for: Severe bacterial neonatal infections in Madagascar, Senegal, and Cambodia: A multicentric community-based cohort study
Source: PLoS Med. 2021 Sep 28;18(9):e1003681. doi: 10.1371/journal.pmed.1003681 (PMC8478182; doi:10.1371/journal.pmed.1003681)
Supplement: S1 Checklist — STROBE, Strengthening the Reporting of Observational Studies in Epidemiology. (DOC) [file pmed.1003681.s001.doc]

STROBE Statement—Checklist of items that should be included in reports of ***cohort studies***

|  | Item No | Recommendation |
| --- | --- | --- |
| **Title and abstract** | 1 | (*a*) Indicate the study’s design with a commonly used term in the title or the abstract  **Title:** “a multicentric community-based cohort study” |
|  |  | (*b*) Provide in the abstract an informative and balanced summary of what was done and what was found  **-Section abstract/ subsection: methods and findings, paragraphs 1-4**  **-Section abstract/ subsection conclusion** |
| Introduction | | |
| Background/rationale | 2 | Explain the scientific background and rationale for the investigation being reported  **Section introduction : Paragraphs 1-5** |
| Objectives | 3 | State specific objectives, including any prespecified hypotheses  **Section introduction : Paragraph 5** |
| Methods | | |
| Study design | 4 | Present key elements of study design early in the paper  **Section methods/ subsection: study areas and design**  **Additional details in supporting information: S1 Text** |
| Setting | 5 | Describe the setting, locations, and relevant dates, including periods of recruitment, exposure, follow-up, and data collection  **Section methods/ subsection: study areas and design** |
| Participants | 6 | (*a*) Give the eligibility criteria, and the sources and methods of selection of participants. Describe methods of follow-up  **Section methods/ subsection: study areas and design** |
|  |  | (*b*) For matched studies, give matching criteria and number of exposed and unexposed **NA** |
| Variables | 7 | Clearly define all outcomes, exposures, predictors, potential confounders, and effect modifiers. Give diagnostic criteria, if applicable  **Section methods/ subsection : case ascertainment**  **Section methods/ subsection : statistical analysis, paragraph 3** |
| Data sources/ measurement | 8* | For each variable of interest, give sources of data and details of methods of assessment (measurement). Describe comparability of assessment methods if there is more than one group  **Section methods/ subsection: study areas and design** |
| Bias | 9 | Describe any efforts to address potential sources of bias  **Section methods/ subsection : study areas and design, paragraph 2**  **Section methods/ subsection : statistical analysis, paragraph 3** |
| Study size | 10 | Explain how the study size was arrived at  **Section methods/ subsection : sample size** |
| Quantitative variables | 11 | Explain how quantitative variables were handled in the analyses. If applicable, describe which groupings were chosen and why  **Section methods/ subsection : case ascertainment, paragraph 5**  **Section methods/ subsection : statistical analysis, paragraph 2**  **Table 1** |
| Statistical methods | 12 | (*a*) Describe all statistical methods, including those used to control for confounding  **Section methods/ subsection : statistical analysis, paragraphs 3-5**  **Additional details in supporting information: S4 Table** |
|  |  | (*b*) Describe any methods used to examine subgroups and interactions  **Section methods/ subsection : statistical analysis, paragraphs 3-5** |
|  |  | (*c*) Explain how missing data were addressed  **Section methods/ subsection : statistical analysis, paragraphs 6**  **Additional details in supporting information: S5 Table** |
|  |  | (*d*) If applicable, explain how loss to follow-up was addressed **NA** |
|  |  | (*e*) Describe any sensitivity analyses  **Section methods/ subsection : statistical analysis, paragraphs 6**  **Additional details in supporting information: S5 Table** |
| Results | | |
| Participants | 13* | (a) Report numbers of individuals at each stage of study—eg numbers potentially eligible, examined for eligibility, confirmed eligible, included in the study, completing follow-up, and analysed  **Section Results: paragraph 1**  **Figure 1** |
|  |  | (b) Give reasons for non-participation at each stage  **Figure 1** |
|  |  | (c) Consider use of a flow diagram  **Figure 1** |
| Descriptive data | 14* | (a) Give characteristics of study participants (eg demographic, clinical, social) and information on exposures and potential confounders  **Section Results: paragraph 1**  **Table 1** |
|  |  | (b) Indicate number of participants with missing data for each variable of interest  **Table 1** |
|  |  | (c) Summarise follow-up time (eg, average and total amount)  **Section Results: paragraph 1**  **Figure 1** |
| Outcome data | 15* | Report numbers of outcome events or summary measures over time  **Section Results/ subsection Incidence of severe neonatal infection**  **Figure 4** |
| Main results | 16 | (*a*) Give unadjusted estimates and, if applicable, confounder-adjusted estimates and their precision (eg, 95% confidence interval). Make clear which confounders were adjusted for and why they were included  **Section Results/ subsection : factors associated with early pSBI**  **Section Methods/ subsection : statistical analysis, paragraph 3**  **Table 2** |
|  |  | (*b*) Report category boundaries when continuous variables were categorized  **Table 2** |
|  |  | (*c*) If relevant, consider translating estimates of relative risk into absolute risk for a meaningful time period **NA** |
| Other analyses | 17 | Report other analyses done—eg analyses of subgroups and interactions, and sensitivity analyses  **Section Results/ subsection : factors associated with early pSBI, paragraphs 2 and 3**  **Additional details in supporting information: S5 Table, S6 Table** |
| Discussion | | |
| Key results | 18 | Summarise key results with reference to study objectives  **Section Discussion : paragraph 1** |
| Limitations | 19 | Discuss limitations of the study, taking into account sources of potential bias or imprecision. Discuss both direction and magnitude of any potential bias  **Section Discussion : paragraph 12-13** |
| Interpretation | 20 | Give a cautious overall interpretation of results considering objectives, limitations, multiplicity of analyses, results from similar studies, and other relevant evidence  **Section Discussion : paragraph 2-11** |
| Generalisability | 21 | Discuss the generalisability (external validity) of the study results  **Section Discussion : paragraph 14** |
| Other information | | |
| Funding | 22 | Give the source of funding and the role of the funders for the present study and, if applicable, for the original study on which the present article is based  **Reported with submission** |

*Give information separately for exposed and unexposed groups.

**Note:** An Explanation and Elaboration article discusses each checklist item and gives methodological background and published examples of transparent reporting. The STROBE checklist is best used in conjunction with this article (freely available on the Web sites of PLoS Medicine at http://www.plosmedicine.org/, Annals of Internal Medicine at http://www.annals.org/, and Epidemiology at http://www.epidem.com/). Information on the STROBE Initiative is available at http://www.strobe-statement.org.
