# Supplementary material for: Severe bacterial neonatal infections in Madagascar, Senegal, and Cambodia: A multicentric community-based cohort study
Source: PLoS Med. 2021 Sep 28;18(9):e1003681. doi: 10.1371/journal.pmed.1003681 (PMC8478182; doi:10.1371/journal.pmed.1003681)
Supplement: S1 Text — (DOCX) [file pmed.1003681.s002.docx]

**S1 Text. Study protocol**

**Study areas**

Madagascar

The study was implemented in 3 urban districts (Avaradoha, Besarety, and Soavinadriana) of Antananarivo (the capital of Madagascar, population 14 997 and 4 128 women of childbearing age) and in the rural city of Moramanga (population 17,159 and 3,795 women of childbearing age).

Cambodia

The study was implemented in 2 communes of Steung Meanchey, an urban district of Phnom Penh (the capital of Cambodia, population 87035 and 18 900 women of childbearing age) and 2 districts (Sophor Thep and Rokha Tom) of Kampong Speu, a rural province located 50 km from Phnom Penh (population 79 000 and 18 517 women of childbearing age).

Senegal

The study was implemented in 2 areas of Wakhinane Nimzatt, an urban commune d’arrondissement of Guédiawaye a city near Dakak (population 20 529) and Sokone a rural city close to the Gambia border (population 14 500).

**Design of the BIRDY program**

1. **Study population and recruitment sources**

The study population consists of neonates followed from birth until the age of two years. Two periods have been identified for recruitment: at birth, or before birth in the so–called “pre-inclusion” phase.

1. **General description of the cohort**

The general organization of the investigation is illustrated in figure 1 and in figure 2.

Figure 1: Recruitment steps

b-Weekly census of pregnant women+Pre-inclusion from the beginning of the 3^rd^ trimester of the pregnancy

c-Inclusion of the child

a-Identification of the population on a geographical basis (n adjacent districts accounting for circa 50 live births per month)

**2.1 Recruitment (see**

Fig1a : Identification of the population on a geographical basis Fig 1b : Identification of pregnant with the help of community healthcare workers. Enrolment of the pregnant women during their third trimester of pregnancy Figure 1c: Inclusion of pregnant women’s newborn at birth.

Pregnant women

At each site, we first organize the exhaustive identification of pregnant women within a geographically based defined population. Each pregnant woman is asked to participate in the study.

Pregnant women are systematically provided with information about the project during prenatal consultations.

Pre-inclusion occurs during third trimester consultation for all women meeting the pre-inclusion criteria (see criteria for inclusion section) and giving informed written consent.

At the time of pre-inclusion, the “maternal” section of the electronic Clinical Report Form (e–CRFm) concerning the socio-demographic, medical, and obstetric characteristics of the mother is completed.

The neonates born from the pre-included mothers are included in the study at the time of delivery.

Given the main objective of the study, which relates to the measurement of the incidence of bacterial infections, and the potentially large number of women who do not have antenatal consultations, we optimize the exhaustiveness of live birth recruitment by also including neonates at the time of birth, without a prior pre-inclusion phase.

Births are much easier to identify for deliveries in healthcare structures participating in this project. However, because of a high proportion of home births, particularly in rural environments, we ensure that women giving birth outside healthcare structures are identified as rapidly as possible, through regular monitoring of mothers around the predicted date of delivery, by using text messages and mobile phones and by involving community workers, particularly in rural environments, and working with the traditional birth attendants.

Investigators seek information daily from healthcare structures and traditional birth attendants participating in the project concerning any births occurring over the last 24 hours.

To help ensure that the recruitment of the children is exhaustive, we also compare our data with the official birth registry where possible.

At birth

Neonates are included in the study if they meet the inclusion criteria (see criteria for inclusion section).

A rectal and a vaginal swab sample is taken in mothers giving birth in a healthcare structure.

The e–CRFm is completed at the time of the delivery if the mother has not already been pre-included, and the neonate is thus recruited at the time of delivery.

At the time of birth, an initial “child” e–CRFc concerning the characteristics of the delivery is completed. Anthropometric measurements (weight, length, head circumference and brachial circumference) are recorded. An APGAR score is obtained by the care staff handling the delivery, one, five, and 10 minutes after delivery. The neonate is examined by a health care worker who checks the presence of risk factors for infection (Screening criteria for suspected infection section).

If the mother gives birth with the assistance of a traditional birth attendant (not in a health care structure) the investigator is notified as soon as possible, for the collection of medical information from the traditional birth attendant concerning the birth and the neonate. The investigator checks the presence of risk factors for infection. Babies at risk for infection are referred to a participating hospital for pediatric evaluation. If the neonate dies before the arrival of the investigator, the e–CRFc is completed and a verbal autopsy is carried out by the investigator with the traditional birth attendant and, if possible, the mother or a relative.

The presence of a risk factor for infection at birth systematically leads to collection of the following samples within few hours after birth: gastric fluid (before the first feed), deep auditory canal swabs, anal swabs and a placental biopsy.

The decision as to whether to administer an empirical antibiotic treatment at birth is made in accordance with WHO criteria. If the decision is made to begin empirical antibiotic treatment, blood cultures and blood sampling for CRP determination is performed beforehand in addition to the collection of the systematic samples. A chest X- ray and lumbar puncture may also be requested, at the discretion of the clinician, according to the clinical context.

1. **Follow–up (see figure 2)**

General organization

After their births the children are followed for the first two years of their lives. Follow-up is both passive and active.

Throughout the follow–up period, passive follow–up consists in asking the mother to contact an investigator whenever the child has fever or meets criteria suggestive of infection (Screening criteria for suspected infection section). An information leaflet describing these criteria is distributed and explained to the mothers beforehand. The mothers are also provided with a thermometer and asked to check the child’s axillary temperature. Body temperature is monitored daily during the first month of life, and then weekly for the rest of the follow–up period. The temperatures are recorded on a paper document (which is adapted appropriately if the mother is unable to read).

Close active follow–up is also carried out to minimize the number of missed or uncharacterized infections. During the first seven days of life, an update concerning the child is requested daily by sending a text message to a mobile telephone. In addition, two home visits are planned, the first one within three days of delivery. During this initial visit, the investigator determines the gestational age of the newborn using “Ballard” score^1^.

Thereafter, routine check–ups take place weekly during the first month of life, then fortnightly until three months of age and monthly between three months and twelve months and then every 2 months between 1 and 2 years of age. These check-ups are carried out by investigators and makes it possible to note the occurrence of infectious episodes that have not already been identified, to detect possible infections at the time of the check-up, to remind the mother of the importance of the continuing participation of her child in the cohort, and to provide useful information for the follow–up of the child. The child is also weighed and measured (height, brachial circumference, and head circumference).

All the investigators participating in this project are trained in techniques for weighing the children, taking anthropometric measurements, determining “Ballard” scores^[[1]](#footnote-2)^ and in the clinical evaluation of infection criteria. Furthermore, regular checks are made to ensure the correct execution of the various measurement techniques. All investigators were also trained to measure the temperature. Battery-operated digital thermometers were used and the temperature was taken under the arm. The thermometer was placed under the armpit after checking that no cloth or sheet was between the thermometer and the armpit. The thermometer was clamped with the upper-arm of the child, while ensuring that the child did not move too much. The thermometer was hold in place until it beeped. The temperature was then obtained by adding 0.5° Celsius to temperature read on the thermometer.

1. **Presence of infection criteria during follow-up (see criteria of infection section)**

A criterion for infection may be detected by the mother, who may then consult at the hospital or the Primary Care Center (PCC), or call the investigator. The temperature chart completed by the mother is shown to the investigator during his or her visit, or to the staff of the health center or hospital in cases of direct consultation.

A criterion for infection may be observed by the investigator during routine check–up visits or during a visit at the request of the mother.

When fever is confirmed (axillary temperature ≥ 37.5C) or in the presence of other clinical criteria for infection, the child is to be examined by a medical doctor (at the reference/district hospital or at the PCC). If the investigator has followed initial basic paramedical or medical training, he or she may determine whether the state of health of the child requires immediate hospitalization or whether the treatment at the PCC is possible. If the investigator has no basic paramedical or medical training, he or she sends the child directly to hospital if a criterion of infection is detected.

The signs and symptoms of the child, the final diagnosis and the samples taken are recorded in the e–CRFc.

The medical evaluation is carried out by the attending medical doctor, who completes a paper questionnaire. At the time of data entry, the investigator checks that the collected information is complete, and adds any missing information, with the assistance of the doctor, when necessary:

- Systematic samples in cases in which clinical criteria for suspected infection are identified: urine samples for cytobacteriological examination; blood for blood cultures; lumbar puncture in febrile children under the age of three months; and thick blood smears if the child has a fever in a malaria-endemic area. Blood formula and C–reactive protein (CRP) determinations are also carried out.
- Samples based on the presence of particular warning signs of infection: stool samples for coproculture in cases of diarrhoea (at least three liquid stools per day); lumbar puncture in the presence of neurological signs or convulsions; swabbing in cases of discharge from the eyes or ears or in cases of signs of omphalitis (swabbing of the pus).

When possible, a chest X-ray is carried out in the presence of respiratory signs, at the discretion of the attending clinician. Samples for bacteriological analysis are transported as rapidly as possible in a cold box encased in secure packaging to the microbiological laboratory of the Institut Pasteur of each site.

Figure 2: Follow-up

Delivery

Pregnancy Week 1 Week 4 Month 3 Month 24

2 weekly visits Monthly visits

Visits visits every two weeks

Pre-inclusion Inclusion End of follow-up

Women Child

e-CRF 1 e-CRF 2

Pregnant women were enrolled during their third trimester of pregnancy and were actively monitored to include their newborn at birth. Surveillance visits of the infants were scheduled following the rhythm indicated in the figure.

**Criteria for inclusion and exclusion**

**Preinclusion of the women during pregnancy**

Preinclusion criteria:

- Routine residence in the study zone of a participating country
- No plans to move away from the study zone during the period of follow-up for the neonate
- Information provided about the way in which the study will be carried out and about the collection of biological samples from the neonate
- No opposition from the pregnant woman to the research being carried out or to the collection of biological samples
- Signed informed consent form.

Exclusion criteria:

- Residence outside the study zone of a participating country
- Plans to move away from the study zone during the follow-up period of the neonate
- No information provided about the way in which the study is carried out or about the collection of biological samples from the neonate
- Opposition from the woman to the research being carried out or to biological samples being collected from the neonate.

**Inclusion of the neonate at delivery**

Inclusion criteria:

- Neonate born to parents living in the study zone of a participating country
- Parents of the neonate not intending to move away from the study zone during the follow-up period
- Legal guardians of the neonate informed about the way in which the study is to be carried out and about the collection of biological samples
- Legal guardians of the neonate having no objection to the collection of biological samples
- Authorization from at least one of the legal guardians of the child, in the form of a signed informed consent form.

Exclusion criteria:

- Stillborn neonate
- Parents of the neonate living outside the study zone of a participating country
- Neonate born to parents planning to move away from the study zone of a participating country during the follow-up period
- At least one of the legal guardians of the neonate not informed about the study or about the collection of biological samples
- At least one of the legal guardians of the neonate opposed to the collection of biological samples.
- Neonate already participating in another biomedical study.

NB: being below the age of majority is not considered a criterion for the non-preinclusion of a mother or her infant. However, in such cases, informed consent must be obtained from the mother herself and from one of her parents or legal guardian

**Screening criteria for suspected infection**

**At birth**

Risk factors for infection at birth leading to perinatal sampling and medical evaluation (risk factors are checked by the traditional birth attendant or the midwife in case of home birth and by the midwife or the attending doctor in case of hospital birth):

- Unplanned preterm delivery (<37 weeks gestation)
- Prolonged membrane rupture (≥ 12 h)
- Maternal fever (axillary temperature >37.9 C) at the time of delivery
- Low birth weight (<2500 g)
- Difficult birth (birth asphyxia)
- Foul-smelling amniotic fluid
- Infection in a twin
- Leukorrhoea or untreated urinary infection during pregnancy
- Home birth

**During the neonatal period (0-28 days):**

Criteria for suspected infection leading to medical evaluation and bacterial sampling (criteria used by healthcare agents):

- Feeding difficulties
- Restlessness, irritability
- Lethargy, movement only when stimulated, hypotonia, coma
- Bulging fontanelle
- Convulsions
- Abdominal distension
- Paleness or grey skin
- Redness around umbilicus or purulent discharge from the umbilicus
- Prolonged capillary refill (> 3s)
- Respiratory rate > 60/min
- Apnoea (>15s) or bradypnoea (respiratory rate <20/min)
- Difficult breathing (grunting or severe chest indrawing)
- Cyanosis
- Hypothermia (axillary temperature < 35.5C)
- Fever (axillary temperature > 37.5C)
- Purulent discharge from the eyes
- Marked jaundice
- Many or severe skin pustules

**Criteria to distinguish true bloodstream infection from contamination due to *Staphylococcal spp***

We considered the *Staphylococcal spp* not a contaminant if the neonates presented:

-at least 3 symptoms among respiratory distress (severe chest indrawing or tachypnea), hypo-(axillary temperature < 35.5C) or hyperthermia (axillary temperature > 37.5C), convulsions, poor feeding, unusual behavior (hypotonia or lethargy or irritability).

-or at least 2 symptoms if the neonate concomitantly had an omphalitis or a soft-tissue infection.

**Flow-chart for diagnosis and care of infants and newborns**

-Vaginal swab (last prenatal consultation)

**Recruitment of mothers**

during pregnancy/last prenatal consultation

-Vaginal swab if not done during pregnancy

-Rectal swab

**Home or hospital birth**

**Presence of WHO risk factors for neonatal infection at birth? ^(1)^**

**Medical evaluation at hospital**

- **Systematic samples**: deep
- auditory canal and anal swab, placenta
- **If foul-smelling amniotic fluid, maternal fever, premature rupture of membranes 24h, vaginal sample + for GBS or GNB**: gastric fluid sampling before first feed
- **As required by the attending physician:** chest X-ray

**yes**

**no**

- **Discharge**

**- Information form given to mothers for home surveillance**

**Clinical reevaluation if samples positive**

**Surveillance by mother or field researcher from birth to 6 months**

Presence of criteria of suspected infection? (See screening criteria for suspected infection)

**Suspicion of neonatal infection at birth according to predefined criteria** (See screening criteria for suspected infection)

**no**

**Discharge**

**yes**

**yes**

**no**

**Continued surveillance**

**- Hospital admission**

**- Systematically before antibiotic treatment:** complete blood count, CRP, blood culture

- **As indicated by the attending physician:** chest X-ray, lumbar puncture

**Medical evaluation and bacterial sampling:**

- Blood culture
- Urinalysis
- Complete blood count and CRP
- Lumbar puncture
  - if fever and <3 months
  - if convulsions and/or neurological sign
- In the presence of localized signs: purulent discharge from eye, ear or umbilicus, stools in case of diarrhoea or dysentery
- Thick and thin blood smears if in endemic area

**Care adapted to the clinical situation** (infection type, symptoms)

Empiric ATBs if suspected bacterial infection

Antibiotic regimen adapted at 48h according to isolated bacteria, antimicrobial susceptibility and clinical evolution

**Microbiological procedures of the BIRDY study**

1. **Blood cultures**

Incubation was done at 35 ± 2°C up to 5 days if an automated device was used or 7 days if a manual procedure was applied. In case of manual procedure and use of a two-phase impregnation bottle, a daily inspection supported by the spread of liquid medium on a solid medium were carried out.
If positive, a direct microscopic examination of the broth and gram staining were performed.

**Cultures**

Inoculation of broth and /or colonies on fresh blood agar and chocolate media under a 10% CO_2_ atmosphere at 35 ± 2°C for 24 hours was done.
If multi-microbial growth occured, then selective media were used.
Identification of isolated colonies was made with API galleries. If *Staphylococcus aureus* was suspected, identification was performed by coagulase and agglutination tests (Pastorex). Susceptibility testing was done according to CASFM guidelines.
**Screening of microorganisms**

Screened bacteria were *Haemophilus influenzae*, *Streptococcus pneumoniae* and *agalactiae*, *Enterococcus* spp., *Staphylococcus aureus* and coagulase negative, *Neisseria meningitidis*, Enterobacteriacea and, non-enterobacteria gram negative bacilli.

1. **Urine cyto-bacteriology tests**

**Macroscopic examination is followed by microscopic examination.**A leukocyte count (/mL) with a Malassez cell (or Nageotte) on homogenized urine (threshold 10^4^/mL) was performed followed by a Gram staining on unspun urine.
**Cultures**

Inoculation on selective media was calibrated with a loop of 10μl. Culture media were incubated at 35 ± 2°C for 24 hours (optionally 48 h).

Identification of isolated colonies was made with API galleries. If *Staphylococcus aureus* was suspected, identification was performed by coagulase and agglutination tests (Pastorex). Susceptibility testing was done according to CASFM guidelines.

**Screening of microorganisms**

Screened bacteria were *Escherichia coli*, *Proteus* spp., *Klebsiella* spp., *Enterobacter* spp., *Citrobacter* spp., *Pseudomonas aeruginosa*, *Acinetobacter* spp., *Enterococcus* spp., *Streptococcus agalactiae*, *Staphylococcus aureus* and *saprophyticus*, *Candida* spp.
Urine specimens were stored at +4 ° C until the release of results.

1. **Cerebral spinal fluid cultures**

Macroscopic examination classified CSF samples in clear, hemorrhagic, cloudy or citrine.
**CSF microscopic examination**Erythrocyte/leukocyte counts were performed after homogenization with a counting chamber (Malassez). Quantitative cytology was done after cytocentrifugation, and gram staining allowed tovisualize the microbial flora.

Soluble antigen detection included *Haemophilus influenzae type B*, *Streptococcus pneumoniae*, *Neisseria meningitidis* (serogroups A, C and W/Y)*, Streptococcus agalactiae* and *Escherichia coli* K1.

**Cultures**Two drops of CSF were spread on a chocolate agar supplemented with polyvitex and a blood agar medium and were inoculated into a Brain-Heart-Infusion (BHI) broth. These inoculated media were incubated at 36 ± 1°C with 10% CO_2_ (except BHI) for 5 days.

Identification of isolated colonies was made with API galleries. If *Staphylococcus aureus* was suspected, identification was performed by coagulase and agglutination tests (Pastorex). Susceptibility testing was done according to CASFM guidelines.
**Screening of microorganisms**

Screened bacteria were *Haemophilus influenzae*, *Streptococcus pneumoniae* and *agalactiae*, *Neisseria meningitidis*, *Escherichia coli* K1, *Klebsiella* spp., *Listeria monocytogenes* (aerobic incubation), *Staphylococcus* spp.

Culture-negative CSF with strong suspicion of meningitis were stored at -80 ° C.

1. **Stools**

A calibrated amount of stools was undertaken at baseline and reproduced throughout the project so as to use semi-quantitative methods in bacteria counts.
Microscopic examination included direct examination and examination after staining with methylene blue and Gram.
**Cultures**

Some media were used systematically, Bromo-Cresol-Purple (BCP), Eosin-Methylene-Blue (EMB), Hektoen, Mueller-Kaufmann broth to screen for pathogenic enterobacteria, mainly *Escherichia coli*, *Salmonella* and *Shigella*.
In case of a bloody stool, a Cefsulodin-Irgasan-Novobiocin (CIN) agar was added.
Based on microscopic examination observation, additional solid media could be used, Blood agar supplemented with nalidixic acid for *Staphylococcus.aureus*, Thiosulfate-Citrate-Bile-Saccharose (TCBS) for *Vibrio* spp., and Chromagar Candida for yeast detection.
Media were incubated at 35 ± 2 ° C (except CIN) for 24 to 48 hours.
Identification of isolated colonies was made with API galleries. If *Staphylococcus aureus* was suspected, identification was performed by coagulase and agglutination tests (Pastorex). Susceptibility testing was done according to CASFM guidelines.

**Serotyping**Salmonella were serotyped according to the Kaufmann-White scheme, *E. coli* O157:H7 and Shigella were serotyped with the appropriate antiserums according to the identified species.

1. **Pus**

Macroscopic examination was followed by microscopic examination which includes Gram staining to detect polymorphonuclear leucocytes and to examine the composition of bacterial flora.
**Cultures**
Pus were spread on chocolate agar, blood agar, Chapman and BCP.
In case of anaerobic suspicion, Schaedler agar and broth were inoculated. Incubation was carried out at 35 ± 2°C with or without CO_2_.
Identification of isolated colonies was made by API galleries. If *Staphylococcus aureus* was suspected, identification was performed by coagulase and agglutination tests (Pastorex). Susceptibility testing was done according to CASFM guidelines.

**Screening of microorganisms**

Screened bacteria were *Staphylococcus aureus*, *Streptococcus β-hemolytic*, *Pseudomonas aeruginosa*, *Escherichia coli*, *Klebsiella* spp., *Enterobacter* spp., Serratia spp., *Acinetobacter* spp., *Alcaligenes faecalis*, *Stenotrophomonas* *maltophilia*, *Neisseria gonorrhoeae*.

1. **Perinatal samples (placental, gastric fluid, deep auditory canal swabs, rectal swab)**

**Microscopic examination by Gram stain.
Cultures**

Samples were inoculated on Blood Agar, chocolate Agar supplemented with Polyvitex (incubated under 10% CO_2_) and on enterobacteria media when influenced by gram staining observation. Monomorphic colonies were identified. Identification of isolated colonies was made with API galleries. If *Staphylococcus aureus* is suspected, identification was performed by coagulase and agglutination tests (Pastorex). Susceptibility testing was done according to CASFM guidelines.

**Screening of microorganisms**

Screened bacteria were Enterobacteriaceae, *Staphylococcus aureus*, β-hemolytic streptococci, *Enterococcus* spp., *Pseudomonas aeruginosa*, *Acinetobacter* spp*.*, *Listeria monocytogenes*

1. The Ballard Maturational Assessment, Ballard Score, is a commonly used technique of gestational age assessment. This involves a clinical examination of the neonate evaluating physical and neurological maturity. It assigns a score to various criteria, the sum of all of which is then extrapolated to the gestational age of the baby. These criteria are divided into Physical and Neurological criteria. This scoring allows for the estimation of gestational age in the range of 26 weeks–40 weeks [↑](#footnote-ref-2)
