# Supplementary material for: Severe bacterial neonatal infections in Madagascar, Senegal, and Cambodia: A multicentric community-based cohort study
Source: PLoS Med. 2021 Sep 28;18(9):e1003681. doi: 10.1371/journal.pmed.1003681 (PMC8478182; doi:10.1371/journal.pmed.1003681)
Supplement: S1 Table — (DOCX) [file pmed.1003681.s003.docx]

**S1 Table. Global (both early and late-onset infections), early and late incidences of culture-confirmed and possible severe neonatal infections according to site**

|  |  | **Madagascar** | | | | **Cambodia** | | | **Senegal** | | |
| --- | --- | --- | --- | --- | --- | --- | --- | --- | --- | --- | --- |
|  |  | n | Incidence  [95% CI] | p | | n | Incidence  [95% CI] | p | n | Incidence  [95% CI] | p |
| **Culture-confirmed severe infections** | | | | | | | | | | | |
| **Global** | | 30 | 15.2  [10.6-21.8] |  | 5 | | 6.5 [2.7-15.6] |  | 7 | 10.2 [4.8-21.3] |  |
| Site | *Urban* | 14 | 18.5  [10.9-31.1] | 0.37 | 1 | | 2.9 [0.4-20.5] | 0.26 | 5 | 12.1 [5.0-29.1] | 0.54 |
|  | *Rural* | 16 | 13.2  [8.1-21.6] |  | 4 | | 9.5 [3.6-25.3] |  | 2 | 7.3 [1.8-29.2] |  |
| **Early-onset infections** | | 24 | 12.3  [8.2-18.3] |  | 3 | | 3.9 [1.3-12.1] |  | 6 | 8.7 [3.9-19.4] |  |
| Site | *Urban* | 10 | 13.2  [7.1-24.5] | 0.77 | - | |  | - | 4 | 9.7 [3.6-25.8] | 0.74 |
|  | *Rural* | 14 | 11.7  [6.7-19.7] |  | 3 | | 7.1 [2.3-22] |  | 2 | 7.3 [1.8-29.1] |  |
| **Late-onset infections** | | 6 | 3.1 [1.4-6.8] |  | 2 | | 2.6 [0.6-10.4] |  | 1 | 1.4 [0.2-10.3] |  |
| Site | *Urban* | 4 | 5.3 [2-14] | 0.16 | 1 | | 2.9 [0.4-20.5] | 0.89 | 1 | 2.4 [0.3-17.2] | - |
|  | *Rural* | 2 | 1.7 [0.4-6.7] |  | 1 | | 2.3 [0.3-16.7] |  | 0 |  |  |
| **Possible severe infections** | | | | | | | | | | | |
| **Global** | | 342 | 196.3  [176.5-218.2] |  | 79 | | 110.1  [88.3-137.3] |  | 51 | 78.3  [59.5-103] |  |
| Site | *Urban* | 110 | 161  [133.5-194] | **0.008** | 45 | | 141.1  [105.4-189] | **0.025** | 40 | 103.6  [76-141.2] | **0.006** |
|  | *Rural* | 232 | 219  [192.6-249,1] |  | 34 | | 85.3  [61-119.4] |  | 11 | 41.5 [23-75] |  |
| **Early-onset infections** | | 201 | 115.9  [101-133] |  | 43 | | 59.9  [44.4-80.8] |  | 41 | 63 [46.3-85.5] |  |
| Site | *Urban* | 77 | 114.1  [91.4-142.5] | 0.86 | 23 | | 72.1  [47.9-108.5] | 0.23 | 30 | 77.7  [54.3-111.1] | 0.07 |
|  | *Rural* | 124 | 117.1  [98.2-138.6] |  | 20 | | 50.2  [32.4-77.8] |  | 11 | 41.5  [23-75] |  |
| **Late-onset infections** | | 141 | 80.9  [68.6-95.4] |  | 36 | | 50.2  [36.2-69.6] |  | 10 | 15.4  [8.3-28.5] |  |
| Site | *Urban* | 33 | 48.3  [34.3-67.9] | **0.001** | 22 | | 69  [45.4-104.8] | **0.04** | 10 | 25.9  [13.9-48.1] | - |
|  | *Rural* | 108 | 102  [84.4.1123.1] |  | 14 | | 35.1  [20.8-59.3] |  | 0 |  |  |
| Early onset infections are defined as cases occurring between 0-6 days and late onset infections as cases occurring between 7-28 days. | | | | | | | | | | | |
