# Supplementary material for: Severe bacterial neonatal infections in Madagascar, Senegal, and Cambodia: A multicentric community-based cohort study
Source: PLoS Med. 2021 Sep 28;18(9):e1003681. doi: 10.1371/journal.pmed.1003681 (PMC8478182; doi:10.1371/journal.pmed.1003681)
Supplement: S2 Table — (DOCX) [file pmed.1003681.s004.docx]

**S2 Table. Pathogens isolated in neonatal culture-confirmed severe infections**

|  | N | **Site**  Urban(n)/Rural (n) | | | **Age at infection (days)** | | | **Sample site**  **(n)** | | **Place of birth** | | | **Low birth weight** | **Resistance to ampicillin,**  **and/or gentamicin $** |
| --- | --- | --- | --- | --- | --- | --- | --- | --- | --- | --- | --- | --- | --- | --- |
|  |  | Madagascar | Cambodia | Senegal | 0-3 | 4-7 | 8-30 |  |  | Health-care facilities(n) | | Home (n) |  |  |
| **Gram positive** | | | | | | | | | | | | | | |
| *Staphylococcus aureus* | 5 | 2/1 | 0/1 | 0/1 | 3 |  | 2 | Blood | 4 | 3 | | 2 | 3 | 1 |
|  |  |  |  |  |  |  |  | Abcess | 1 |  |  |  |  |  |
| Staphylococcus species | 1 | 0/1 | 0/0 | 0/0 | 1 |  |  | Blood | 1 | 0 | | 1 | 0 | 0 |
| *Staphylococcus epidermidis* | 4 | 1/2 | 0/1 | 0/0 | 3 |  | 1 | Blood | 4 | 3 | | 1 | 3 | 1 |
| *Staphylococcus haemolyticus* | 1 | 0/0 | 0/0 | 1/0 | 1 |  |  | Blood | 1 | 1 | | 0 | 0 | 1 |
| *Streptococcus pneumoniae* | 1 | 1/0 | 0/0 | 0/0 |  |  | 1 | Blood | 1 | 0 | | 1 | 0 | 1 |
| *Enterococcus faecalis* | 1 | 1/0 | 0/0 | 0/0 |  | 1 |  | Urine | 1 | 1 | | 0 | 1 | 1 |
| **Gram Negative** | | | | | | | | | | | | | | |
| *Klebsisella pneumoniae* (1) (3) | 9 | 3/3 | 0/2 | 1/0 | 4 | 4 | 1 | Blood | 4 | 7 | | 2 | 4 | 7 |
|  |  |  |  |  |  |  |  | Urine | 2 |  |  |  |  |  |
|  |  |  |  |  |  |  |  | Umbilic purulent discharge | 3 |  |  |  |  |  |
| *Klebsiella Oxytoca* | 2 | 2/0 | 0/0 | 0/0 | 1 | 1 |  | Blood | 1 | 2 | | 0 | 0 | 1 |
|  |  |  |  |  |  |  |  | Urine | 1 |  |  |  |  |  |
| *Escherichia coli* (2) | 10 | 3/4 | 1/0 | 1/1 | 5 | 4 | 1 | Blood | 4 | 6 | | 4 | 2 | 9 |
|  |  |  |  |  |  |  |  | Urine | 4 |  |  |  |  |  |
|  |  |  |  |  |  |  |  | Umbilic purulent discharge | 1 |  |  |  |  |  |
|  |  |  |  |  |  |  |  | Cerebrospinal fluid | 1 |  |  |  |  |  |
|  | N | **Site**  Urban(n)/Rural (n) | | | **Age at infection** | | | **Sample site**  **(n)** |  | **Place of birth** | | | **Low birth weight** | **Resistance to ampicillin,**  **and/or gentamicin $** |
|  |  |  |  |  |  |  |  |  |  | Healthcare facilities(n) | | Home (n) |  |  |
|  |  | Madagascar | Cambodia | Senegal | 0-3 | 4-7 | 8-30 |  |  |  |  |  |  |  |
| *Enterobacter cloacae* (1) (2) | 3 | 0/2 | 0/0 | 1/0 | 1 | 1 | 1 | Blood | 2 | 1 | 2 | | 1 | 1 |
|  |  |  |  |  |  |  |  | Umbilic purulent discharge | 1 |  |  |  |  |  |
| *Acinetobacter Baumanii* | 3 | 1/1 | 0/0 | 1/0 | 2 |  | 1 | Blood | 2 | 2 | 1 | | 1 | 0 |
|  |  |  |  |  |  |  |  | Umbilic purulent discharge | 1 |  |  |  |  |  |
| *Pseudomonas aeruginosa* (3) | 1 | 0/0 | 0/0 | 1/0 |  | 1 |  | Umbilic purulent discharge | 1 | 1 | 0 | | - | 0 |
| Pasteurella | 1 | 0/1 | 0/0 | 0/0 |  |  | 1 | Cerebrospinal fluid | 1 | 1 | 0 | | 1 |  |
| Gram negative staining* | 3 | 1/2 | 0/0 | 0/0 | 3 |  |  | Cerebrospinal fluid | 3 | 2 | 1 | | 0 |  |

$ Resistance to ampicillin was not taken in account for Klebsisella pneumoniae and Enterobacter cloacae as these pathogens are naturally resistant to ampicillin

*Pathogens identified by staining as gram-negative but could not be cultured and, therefore, were not further identified.

(1) one Klebsisella pneumoniae and one Enterobacter cloacae were found on the same umbilic purulent discharge sample, (2) one E coli and one Enterococcus faecalis on the same urine sample, (3) one Klebsisella pneumoniae and one Pseudomonas aeruginosa umbilic on the same umbilic purulent discharge sample.
