# Supplementary material for: Severe bacterial neonatal infections in Madagascar, Senegal, and Cambodia: A multicentric community-based cohort study
Source: PLoS Med. 2021 Sep 28;18(9):e1003681. doi: 10.1371/journal.pmed.1003681 (PMC8478182; doi:10.1371/journal.pmed.1003681)
Supplement: S3 Table — (DOCX) [file pmed.1003681.s005.docx]

**S3 Table. Susceptibility pattern of isolated bacteria in culture-confirmed neonatal infections**

|  | **AMP** | | **AMC** | | **TIC** | | | **GEN** | | | **AMK** | | **TZP** | | **SXT** | | **CPH** | | **FOX** | | **CTX** | | **CAZ** | | **CIP** | | **ERY** | | **IMP** | | **CHL** | | **TET** | | **OXA** | | **VAN** | | **TEC** | |
| --- | --- | --- | --- | --- | --- | --- | --- | --- | --- | --- | --- | --- | --- | --- | --- | --- | --- | --- | --- | --- | --- | --- | --- | --- | --- | --- | --- | --- | --- | --- | --- | --- | --- | --- | --- | --- | --- | --- | --- | --- |
|  | n | R | n | R | n | | R | n | R | | n | R | n | R | n | R | n | R | n | R | n | R | n | R | n | R | n | R | n | R | n | R | n | R | n | R | n | R | n | R |
| **Gram positive** | | | | | | | | | | | | | | | | | | | | | | | | | | | | | | | | | | | | | | | | |
| *Staphylococcus aureus* |  | |  | |  | | | 5 | | 1 |  | |  | | 5 | 1 |  | | 5 | 1 |  | |  | | 5 | 0 | 5 | 1 |  | | 4 | 0 | 4 | 0 |  | | 4 | 0 | 4 | 0 |
| *Staphylococcus epidermidis* |  | |  | |  | | | 4 | | 1 |  | |  | | 4 | 3 |  | | 4 | 1 |  | |  | | 3 | 0 | 4 | 0 |  | | 3 | 1 | 4 | 1 |  | | 4 | 0 | 4 | 0 |
| *Staphylococcus haemotylicus* |  | |  | |  | | | 1 | | 1 |  | |  | | 1 | 1 |  | | 1 | 1 |  | |  | | 1 | 1 | 1 | 1 |  | | 1 | 0 | 1 | 1 |  | |  |  |  |  |
| *Staphylococcus species* |  | |  | |  | | | 1 | | 0 |  | |  | | 1 | 1 |  | | 1 | 0 |  | |  | | 1 | 0 | 1 | 0 |  | |  |  | 1 | 1 |  | | 1 | 0 | 1 | 0 |
| *Streptococcus pneumoniae* | 1 | 0 |  | |  | | | 1 | | 1 |  | |  | | 1 | 0 |  | |  | |  | |  | | 1 | 0 | 1 | 0 |  | | 1 | 0 | 1 | 1 | 1 | 0 | 1 | 0 | 1 | 0 |
| *Enterococcus faecalis* | 1 | 0 |  | |  | | | 1 | | 1 |  | |  | | 1 | 0 |  | |  | |  | |  | |  |  | 1 | 1 |  | | 1 | 0 | 1 | 0 | 1 | 1 | 1 | 0 | 1 | 0 |
| **Gram negative** | | | | | | | | | | | | | | | | | | | | | | | | | | | | | | | | | | | | | | | | |
| *Klebsiella pneumoniae* | 9 | 9 | 9 | 6 | 7 | | 7 | 9 | | 7 | 9 | 0 | 7 | 1 | 6 | 6 | 8 | 5 | 7 | 0 | 9 | 6 |  | | 9 | 4 |  | | 8 | 0 | 8 | 3 |  | |  | |  | |  | |
| *Klebsiella oxytoca* | 2 | 2 |  | | 2 | | 2 | 2 | | 1 |  |  | 2 | 0 | 2 | 2 | 2 | 2 | 2 | 1 | 2 | 2 |  | | 2 | 1 |  | | 2 | 0 | 2 | 2 |  | |  | |  | |  | |
| *Escherichia coli* | 10 | 9 | 10 | 4 | 10 | | 9 | 10 | | 4 | 9 | 0 | 8 | 2 | 10 | 7 | 9 | 2 | 9 | 0 | 10 | 3 |  | | 10 | 3 |  | | 9 | 0 | 8 | 0 |  | |  | |  | |  | |
| *Enterobacter cloacae* | 3 | 3 | 3 | 3 | 3 | | 2 | 3 | | 1 | 3 | 0 | 3 | 1 | 1 | 0 | 3 | 3 | 3 | 3 | 3 | 2 |  | | 3 | 2 |  | | 2 | 0 | 3 | 0 |  | |  | |  | |  | |
| *Pasteurella spp.* |  | |  | |  | | |  | | |  | |  | |  | |  | |  | | 1 | 0 |  | | 1 | 0 |  | |  |  |  |  | 1 | 0 |  | |  | |  | |
| *Acinetobacter baumanii* |  | | TIM | | 3 | 1 | | 3 | | 0 | 3 | 0 |  | | 3 | 1 |  | |  | |  | | 3 | 2 | 3 | 0 |  | | 3 | 0 | 2 | 2 |  | |  | |  | |  | |
|  |  |  | 2 | 1 |  |  |  |  |  |  |  |  |  |  |  |  |  |  |  |  |  |  |  |  |  |  |  |  |  |  |  |  |  |  |  |  |  |  |  |  |
| *Pseudomonas aeruginosa* |  | | TIM | | 1 | 0 | | 1 | | 0 | 1 | 0 | 1 | 0 |  | |  | |  | | FEP | | 1 | 0 | 1 | 0 |  | |  | |  | |  | |  | |  | |  | |
|  |  |  | 1 | 0 |  |  |  |  |  |  |  |  |  |  |  |  |  |  |  |  | 1 | 0 |  |  |  |  |  |  |  |  |  |  |  |  |  |  |  |  |  |  |
| **n : total number of isolates R number of resistant isolates**  **AMP**: ampicillin, **AMC:** amoxicillin-clavulanic acid, **TIC**: ticarcillin, **GEN**: gentamycin, **AMK**: amikacin, **TZP**: piperacillin-tazobactam, **SXT**: trimethoprim-sulfamethoxazole, **CPH**: cefalotin, **FOX**: cefoxitin, **CTX:** cefotaxime, **CAZ**: ceftazidime, **CIP**: ciprofloxacin, **ERY**: erythromycin, **IPM**: imipenem, **CHL**: chloramphenicol, **TET**: tetracycline, **OXA**: oxacillin, **VAN**: vancomycin, **TEC**: teicoplanin, **TIM**: ticarcillin-clavulanic acid, **FEP**: cefepime | | | | | | | | | | | | | | | | | | | | | | | | | | | | | | | | | | | | | | | | |
