# Supplementary material for: Severe bacterial neonatal infections in Madagascar, Senegal, and Cambodia: A multicentric community-based cohort study
Source: PLoS Med. 2021 Sep 28;18(9):e1003681. doi: 10.1371/journal.pmed.1003681 (PMC8478182; doi:10.1371/journal.pmed.1003681)
Supplement: S4 Table — (DOCX) [file pmed.1003681.s006.docx]

**S4 Table. Risk factors analysis of possible severe bacterial neonatal infection in Cambodia with parametric regression model, with an accelerated failure time approach**

| **Cambodia N=769** | | | | |
| --- | --- | --- | --- | --- |
|  | Crude time ratio [95% CI] | p | Adjusted time ratio^a^ [95% CI] | p |
| **Site (urban site as reference)** | 1.03 [0.63-1.7] | 0.89 |  |  |
| **Education** |  |  |  |  |
| Absence/  primary school | Ref |  |  |  |
| Partial secondary school | 1.24  [0.72-2.14] | 0.45 |  |  |
| Complete secondary or higher | 1.56  [0.68-3.57] | 0.29 |  |  |
| **Primigravidae** | 0.79  [0.47-1.31] | 0.36 |  |  |
| **Twins pregnancy** | 0.03 [0.01-0.11] | 0.001 |  |  |
| **Hospitalization during pregnancy** | 1.45  [0.29-7.16] | 0.65 |  |  |
| **Skilled birth attendant** | -^b^ |  |  |  |
| **Sex of newborn (boys as reference)** | 1.1 [0.67-1.8] | 0.7 |  |  |
| **Delivery in health care facilities** | -^b^ |  |  |  |
| **Low birth weight** | 0.12  [0.06-0.26] | <0.001 | 0.11  [0.05-0.24] | <0.001 |
| **Cesarean -section** | 0.8  [0.4-1.61] | 0.53 |  |  |
| **Fetid amniotic fluid** | 0.23  [0.07-0.75] | 0.01 | 0.25  [0.09-0.69] | 0.008 |
| **Dystocic delivery** | 0.49 [0.18-1.31] | 0.16 |  |  |

^a^ Adjusted on site (aHR=0.99 [0.62-1.6], p=0.9] and sex of the newborn (aHR=1.2 [0.76-1.9], p=0.4)

^b^No possible estimation as no cases of pSBI occurred among neonates who delivered out of health-care facilities and among those who delivered with unqualified health-care workers

To document the direction and strength of “low birth weight” association with pSBI, we here present a parametric model. The distribution of the outcome was first assessed visually and was compatible with both a log-normal distribution and a log-logistic distribution, which are the distributions that do not require the validity of the proportional hazard assumption. Then, two parametric models with an accelerated failure time approach were performed : one with a log-normal distribution and the other one with a log-logistic distribution. Based on Akaike’s Information Criterion (AIC), the log-normal distribution (AIC=566) better fit the data than the log-logistic distribution (AIC=577) justifying to retain the log-normal distribution. In the adjusted analysis, in neonates with low birth weight, time to onset of severe bacterial infection was about 10 times shorter than in neonates with normal birth weight (adjusted time ratio (aTR): 0.11, p<0.001). In neonates born from mother with foul-smelling amniotic fluid, time to onset of severe bacterial infection was 4 times shorter than in those born from mother without foul-smelling amniotic fluid (aTR: 0.25, p=0.008). In other words, being low birth weight or being born to a mother with foul-smelling amniotic fluid sped-up time to severe bacterial infection.
