# Supplementary material for: Severe bacterial neonatal infections in Madagascar, Senegal, and Cambodia: A multicentric community-based cohort study
Source: PLoS Med. 2021 Sep 28;18(9):e1003681. doi: 10.1371/journal.pmed.1003681 (PMC8478182; doi:10.1371/journal.pmed.1003681)
Supplement: S5 Table — pSBI, possible severe bacterial infection. (DOCX) [file pmed.1003681.s007.docx]

**S5 Table.** Sensitivity analysis: Risk factors analysis of possible severe bacterial infection in Senegal

|  | Worst case scenario |  |  |  | Best case scenario |  |  |  |
| --- | --- | --- | --- | --- | --- | --- | --- | --- |
|  | OR  [95% CI] | p | Adjusted OR^a^ (aOR)  [95% CI] | p | OR  [95% CI] | p | aOR^b^  [95% CI] | p |
| **Site (urban site as reference)** | 0.62  [0.33-1.18] | 0.15 |  |  | 0.62  [0.33-1.18] | 0.15 |  |  |
| **Education** |  |  |  |  |  |  |  |  |
| Absence/  primary school | Reference |  |  |  | Reference |  |  |  |
| Partial secondary school | 0.97  [0.43-2.2] | 0.9 |  |  | 0.97  [0.43-2.2] | 0.9 |  |  |
| Complete secondary or higher | 1.61  [0.68-3.87] | 0.28 |  |  | 1.61  [0.68-3.87] | 0.28 |  |  |
| **Primigravidae** | 1.27  [0.58-2.25] | 0.7 |  |  | 1.27  [0.58-2.25] | 0.7 |  |  |
| **Twins pregnancy** | - ^c^ |  |  |  | - ^c^ |  |  |  |
| **Hospitalization during pregnancy** | 0.73  [0.1-5.28] | 0.76 |  |  | 0.73  [0.1-5.28] | 0.76 |  |  |
| **Skilled birth attendant** | 0.7  [0.1-4.9] | 0.7 |  |  | 0.7  [0.1-4.9] | 0.7 |  |  |
| **Sex of newborn (boys as reference)** | 0.9  [0.5-1.6] | 0.7 |  |  | 0.9  [0.5-1.6] | 0.7 |  |  |
| **Delivery in health care facilities** | 0.68  [0.1-4.9] | 0.7 |  |  | 0.68  [0.1-4.9] | 0.7 |  |  |
| **Low birth weight** | 2.49  [1.15-5.37] | 0.02 | 1.5  [0.7-3] | 0.29 | 2.49  [1.15-5.37] | 0.02 | 2.6  [1.2-5.5] | 0.016 |
| **Cesarean -section** | 1.64  [0.09-4.63] | 0.66 |  |  | 1.64  [0.09-4.63] | 0.66 |  |  |
| **Fetid amniotic fluid** | 2.7  [1.5-4.8] | 0.001 | 2.4  [1.3-4.5] | 0.005 | 3.4  [1.9-6.3] | <0.001 | 3.9  [2.1-7.1] | <0.001 |
| **Dystocic delivery** | 1.25  [0.6-2.59] | 0.56 |  |  | 2.7  [0.67-11.4] | 0.15 |  |  |

^a^ Adjusted on site (aHR=0.7 [0.35-1.3], p=0.24) and sex of the newborn (aHR=0.93[0.52-1.68], p=0.82)

^b^ Adjusted on site (aHR=0.5[0.26-0.97], p=0.04) and sex of the newborn (aHR=0.98[0.55-1.77], p=0.96)

^c^ No possible estimation as no cases of pSBI occurred among twins

We investigated if low birth weight and factors related to delivery (foul-smelling amniotic fluid, premature rupture of the membranes, dystocic delivery, caesarean section) were associated with early pSBI and considered site, sex of the newborns and maternal and pregnancy characteristics as potential confounding factors.

For worst scenario, infants were classified as infants born to mothers with foul-smelling amniotic fluid and/or premature rupture of membranes and/or dystocic delivery depending on which variable was missing. Where as in best scenario, infants were classified as infants as infants born from mother with no foul liquid amniotic fluid and/or no premature rupture of membranes and/or who experienced eutocic delivery.
